# Functional characterization of duodenal microbiota and associated enteropathy in undernourished Bangladeshi women and gnotobiotic mice

**DOI:** 10.64898/2026.08.14.26360472

**Published:** 2026-09-04

**Authors:** Kali M. Pruss, ZeNan L. Chang, Md. Shabab Hossain, M. Masudur Rahman, Mustafa Mahfuz, Reyan Coskun, Rumana Sharmin, A.H.M. Rezwan, Shafiqul Alam Sarker, Subhasish Das, Shah Mohammad Fahim, Md. Amran Gazi, Kelsey A. Hudson, Athziri Marcial Rodríguez, Haoxin Liu, Richard Kitchen, Alexandra E. Byrne, Clara Kao, Brooks B. Brodrick, Abbey Rose, Bishan Bhattarai, Darya Khantakova, Jose Fachi, Marco Colonna, Tahmeed Ahmed, Michael J. Barratt, Jeffrey I. Gordon

**Affiliations:** 1 The Edison Family Center for Genome Sciences and Systems Biology, Washington University School of Medicine; St. Louis, MO 63110, USA.; 2 The Newman Center for Gut Microbiome and Nutrition Research, Washington University School of Medicine; St. Louis, MO 63110, USA.; 3 Division of Gastroenterology, Department of Medicine, Washington University School of Medicine; St. Louis, MO 63110.; 4 International Center for Diarrhoeal Disease Research Bangladesh (icddr,b); Dhaka 1212, Bangladesh.; 5 Department of Gastroenterology, Dhaka Medical College; Dhaka-1000, Bangladesh.; 6 Department of Gastrointestinal, Hepatobiliary and Pancreatic Disorders, Bangladesh Specialized Hospital; Dhaka-1207, Bangladesh.; 7 Division of Immunobiology, Department of Pathology and Immunology, Washington University School of Medicine; St. Louis, MO 63110, USA.

## Abstract

Undernutrition is an intergenerational global health challenge. Environmental enteric dysfunction (EED) is a small intestinal (SI) disorder characterized by villous atrophy, gut barrier dysfunction, malabsorption and systemic inflammation. To examine its pathogenesis and role in undernutrition, we performed esophagogastroduodenoscopy on undernourished Bangladeshi women with EED and their healthy counterparts. Histologic characterization of duodenal mucosal biopsies, aptamer-based proteomic analyses of their duodenal mucosa and plasma, plus metagenomic analyses of their duodenal and fecal microbiota, revealed associations between bacterial taxa and duodenal tissue and plasma proteomes indicative of EED. Colonization of germ-free female mice with consortia of cultured duodenal bacteria from these women, followed by measurements of SI bacterial abundances, SI cellular patterns of gene expression (single nucleus RNA-seq), plus proteomic and flow cytometric analyses disclosed bacterial, epithelial, and immune features of EED in dams and their offspring resembling those in the women. These findings have diagnostic and therapeutic implications.

## One Sentence Summary

Bacteria-protein associations in the small intestine underlying pathogenesis of environmental enteric dysfunction in undernourished Bangladeshi women.

## INTRODUCTION

Undernutrition in children, defined anthropometrically by impaired linear growth (stunting) and/or by impaired ponderal growth (wasting) affects ~200 million children under age five globally, with the greatest burden of disease in the global South (1–4). Moreover, undernutrition is an intergenerational problem with maternal undernutrition being a key predictor of undernutrition in their offspring (4–6). Environmental enteric dysfunction (EED) is a subclinical small intestinal disorder characterized by villous blunting with accompanying reduction in epithelial nutrient absorptive area, mucosal barrier dysfunction and local as well as systemic inflammation (7–13). The true prevalence of EED, its pathogenesis and its contribution to intergenerational undernutrition remain poorly understood, in part due to a paucity of serum and fecal biomarkers validated by concurrent histopathologic examination of small intestinal mucosal biopsies (7).

We previously studied a cohort of children residing in an urban slum located in the Mirpur district of Dhaka, Bangladesh who were stunted or at risk for stunting and failed a standard nutritional intervention for linear growth faltering (14, 15). These children were part of the Bangladeshi EED study (BEED) [ClinicalTrials.gov number, NCT02812615], where informed consent was obtained for esophagogastroduodenoscopy (EGD) to determine whether their duodenal mucosal biopsies showed evidence of EED and where their duodenal microbiota and duodenal mucosal proteome could be characterized. 94% (85/90) of these children had histopathologic evidence of EED (14). For ethical reasons, EGD could not be performed on healthy children living in the same locale.

We cultured bacteria from the duodenal aspirates obtained from children in the BEED study and assembled distinct consortia that were introduced into germ-free adult female (16). Subsequent dam-to-pup transmission of these consortia results revealed how components of the duodenal microbiota associated with EED can produce local and systemic inflammatory responses in adult female gnotobiotic mice as well as impaired prenatal and postnatal growth in their offspring. Moreover, signaling pathways related to intestinal epithelial cell renewal, barrier integrity and immune function were altered in pups (16). This provided preclinical evidence for a causal relationship between the duodenal microbiota and EED in children.

EED in adults has been understudied. A recent case-control observational study described the histopathology of duodenal mucosal biopsies obtained at the time of EGD of undernourished (BMI<18.5 kg/m^2^) adult women residing in Mirpur; 75% exhibited histopathological evidence of EED, including villus atrophy, crypt hyperplasia and immunoinflammatory changes (17–19). The availability of a cohort of healthy women living in the same locale as those who were undernourished provided an opportunity to address questions related to the pathogenesis of EED. Specifically, are there duodenal and plasma proteomic features that are discriminatory for undernourished women with histopathologic evidence of EED versus their healthy counterparts? How do these features correlate with the representation of microbial taxa in their duodenal and fecal microbiota? Can gnotobiotic mice be used to test whether observed microbial features are causally related to EED? Finally, to what extent are these discriminatory features also found in children with EED?

To address these questions, in the current study we perform EGD on cohorts of well-nourished Bangladeshi women living in Dhaka, determined the extent to which their duodenal biopsies had histopathologic features of EED, and then compared their duodenal mucosal and plasma proteomes as well as their duodenal and fecal microbiota to those of undernourished women with EED. Application of multi-omic analytic methods to these biospecimens allowed us to identify significant associations between the abundances of duodenal bacterial taxa and duodenal proteins that distinguished women with EED from their healthy counterparts as well as candidate disease biomarkers present in their plasma. Bacteria cultured from duodenal aspirates obtained from these undernourished and well-nourished women were used to colonize germ-free adult female mice. Transcriptional and proteomic analyses of adult female mice and their offspring revealed links between duodenal bacteria and enteropathy as well as growth faltering that were reflective of EED in women and children. Finally, duodenal bacteria that were associated with immuno-inflammatory responses in our gnotobiotic mouse experiments and in the duodenal mucosal proteome of women were compared to analogous samples from children with EED to further refine our understanding of organisms associated with enteropathy.

## RESULTS

### Study design

The design, recruitment, and exclusion criteria for women living in Mirpur for the BEED study are described in detail elsewhere (15, 17). Briefly, undernourished (BMI<18.5 kg/m^2^) women, 18–45 years old, were recruited for nutritional therapy. Those whose BMI failed to improve (<10% increase) after three months of the nutritional intervention (see Methods) underwent EGD for collection of duodenal mucosal biopsies and duodenal luminal aspirates. Fecal samples and plasma were collected concurrently from 22 women (table S1A). As part of the BEED study, comparably aged, well-nourished women (BMI=18.5–30 kg/m^2^) residing in Mirpur who were seeking treatment for dyspepsia and were eligible for EGD underwent the procedure, had normal endoscopic findings, and thus were diagnosed as having functional dyspepsia. Duodenal biopsies and aspirates were collected from these individuals during EGD (n=33, Fig. 1A,B). Only one of these 33 women had no histopathologic evidence of EED (defined as a score of 0 according to the grading scale described in Methods and refs. (17, 18), table S1A). Therefore, we recruited a second cohort of well-nourished women from Dhaka who were also 18–45 years old. They had BMIs of 20–25 kg/m^2^ and had sought treatment for functional dyspepsia. These women (n=25) underwent EGD and were diagnosed with functional dyspepsia as above. Duodenal aspirates and mucosal biopsies were collected during EGD of these women with concurrent collection of plasma and fecal specimens; 19 had biopsies with histopathology score of 0 (table S1A). Members of the low BMI cohort with histopathologic evidence of EED, and the two well-nourished cohorts (with or without evidence of EED) were characterized using plasma proteomics (SomaScan 11k arrays), duodenal mucosal proteomics (SomaScan 7k arrays), and duodenal and fecal microbiota profiling (full-length bacterial 16S rRNA amplicon sequencing).

### Plasma and duodenal proteomes of undernourished women with EED

Dimensionality reduction (sparse partial least squares discriminant analysis, sPLS-DA) (20) showed that protein composition within the duodenal mucosa separated primarily by histopathology score (Fig. 1C). Levels of 1,042 duodenal proteins (1,068 unique aptamers) were significantly different between women with EED (histopathology score 1–3, n=59) compared to those without EED (score 0, n=20, *limma* adjusted *P*-value<0.05; table S1B). 103 Reactome pathways were significantly enriched in the duodenal mucosal proteomes of women with EED; they included several pathways related to immune activation (‘TNF signaling’, ‘Interleukin-17 signaling’, ‘Toll Like Receptor Cascades’, ‘MAP kinase activation’, ‘Oxidative Stress Induced Senescence’, ‘Death Receptor Signaling’, and ‘Cellular response to starvation’, fig. S1A, table S1B).

To identify potential non-invasive biomarkers of EED, we cross-referenced the set of duodenal proteins that were elevated in women having histopathology scores ≥1 (676 proteins) with plasma proteins that were negatively correlated with BMI (i.e., proteins that had higher levels in undernourished women). A Significance Analysis of Microarray (SAM) analysis identified 629 proteins that were negatively correlated with BMI (640 unique aptamers, FDR<0.05, fig. S1B); they included several complement components (C3, C4a, C1q), pro-inflammatory cytokines and their receptors (IL-17F, IL-18, IL-36, IL-22R, IL-21R, IL-2 and IL-2R), and acute phase proteins (SAA) (fig. S1C, table S1C). Of these 629 plasma proteins, 84 were significantly higher in duodenal biopsies obtained from women with EED. Fig. 1D and table S1D describe in detail the functions of these proteins, which are involved in immune signaling, bacterial sensing, cell adhesion, regulation of cell proliferation and mitochondrial function.

### Associations between duodenal bacterial strain abundances and duodenal mucosal proteins

We conducted full-length 16S rRNA amplicon sequencing of DNA isolated from duodenal aspirates recovered from 22 women in the Low BMI cohort with histopathologic evidence of EED and the 58 well-nourished women (fig. S1D). The absolute abundances of 38 bacterial taxa significantly increased or decreased with histopathology score (zero-inflated Gaussian mixture model *P*-adj<0.1; Methods); they included organisms previously associated with the oral microbiota (Fig. 2A, table S2A–C; see Supplementary Results and table S3A,B for a supervised learning analysis identifying bacteria in aspirates and proteins in biopsies that together predicted disease status).

We next performed linear regressions on the absolute abundances of bacteria and duodenal mucosal proteins to find bacteria-protein pairs whose slopes were significantly different between women with and without histopathologic evidence of EED (Fig. 2B). The bacterial taxa whose abundances exhibited the greatest number of significant associations with levels of duodenal mucosal proteins were *Filifactor alocis* followed by *Oribacterium sp000160135*, *Ligilactobacillus curvatus* (which also significantly increased by histopathology score, Fig. 2A), *Actinomyces massilensis* and *Prevotella melanogenica* (Fig. 2C). The absolute abundances of these bacteria were positively linearly related with a large set of innate immune signaling proteins in adult women with EED, while negatively or not significantly associated with these same proteins in healthy women; they include Sialic Acid-binding Ig-like Lectins (SIGLEC5, SIGLEC6, SIGLEC12, SIGLEC14), Interleukins (IL6, IL7, IL9 IL10, IL12A/B, IL17A/B, IL25, IL31, IL36G) and their receptors (IL1R1/R2, IL3RA, IL4R, IL5RA, IL6R, IL17RD, IL18RAP, IL20RA, IL22RA2), Type I Interferons (Interferon (IFN)A1, A4, A7, A21), components of the complement system (C1QC, C1QL3, C1QTNF9, C3, C5), pathogen sensors (Toll-like receptor 4, TLR4; Peptidoglycan Recognition Protein 1, PGLYRP1, Regenerating Family Member 3, REG3A, REG3G; LysM Domain Containing 3, LYSMD3; C-Type Lectin Domain Family 2 Member A, CLEC2A, CLEC6A, CLEC12A) and chemokines (C-C Motif Chemokine Ligand 7, CCL7; C-X-C Motif Chemokine Ligand 2, CXCL2, CXCL3, CXCL9; Granzyme B, GZMB) (table S3C). An enrichment analysis of the Gene Ontology (GO) categories, KEGG and Reactome pathways disclosed that these proteins are associated with leukocyte migration, cytokine signaling and response to growth factors (fig. S2A, table S3D). Conversely, functions enriched among duodenal mucosal proteins whose levels were significantly negatively associated with the abundances of duodenal bacterial taxa in women with EED included metabolic pathways and responses to steroid hormones (fig. S2B, table S3E). Together, these results reveal associations between duodenal bacterial taxa and duodenal mucosal proteins that are discriminatory for disease in undernourished Bangladeshi women with EED.

#### Fecal microbiota –

We performed comparable analyses on the fecal and plasma specimens obtained from undernourished women and their healthy counterparts (Supplementary Results, tables S4A,B and S5A–E). While 29 exact sequence matches and 203 bacterial species were present in both duodenal aspirates and feces, *Streptococcus thermophilus* was the only bacterium whose abundance was significantly higher in both the duodenal aspirates of women without EED (histopathology scores of 0) and feces of women with healthy BMIs (20–25 kg/m^2^). A TaqMan Array Card qPCR-based assay (21) of DNA extracted from fecal samples revealed that women in the low BMI cohort harbored a greater overall burden of enteric pathogens (Supplementary Results, table S5F), consistent with previous reports in other populations suggesting that subclinical pathogen exposure contributes to pathophysiology of EED and undernutrition (22–24).

### A preclinical test of causality

We next used gnotobiotic mice to directly test whether members of the duodenal microbiota from women with EED could produce mucosal and systemic inflammation. To do so, we cultured and arrayed bacterial isolates from duodenal aspirates. A total of 480 isolates representing 53 unique species (defined from analyses of full length 16S rRNA amplicons) were obtained from 14 undernourished women with EED and 125 isolates representing 35 unique species were recovered from 11 healthy women (BMI, 19–25 kg/m^2^ without histologic evidence of EED) (fig. S3, **table S6A,B**).

We pooled the 480 isolates from the undernourished cohort and the 125 isolates from aspirates obtained from the well-nourished donors. These pooled culture collections were introduced to recently weaned (4.5-week-old) germ-free mice fed a diet representative of that consumed by adults in Mirpur (‘Adult Mirpur’ diet (16)). Eighteen days after gavage of these pools, we measured two serum biomarkers of systemic inflammation that we had identified in children with EED (14). We also demonstrated that these proteins were robust biomarkers of intestinal and systemic inflammation in a gnotobiotic mouse model of dam-to-pup transmission of bacteria cultured from children with EED enrolled in the BEED study (16). Although levels of LCN2 and MMP8 in the sera of mice colonized with the pooled culture collections from the two groups of women were not significantly different after 18 days (fig. S4A, table S7A), we clarified cecal contents from (i) individual mice that had been colonized with the EED donor-derived consortium with the highest levels of serum LCN2 and (ii) from individual animals that had been colonized with the well-nourished donor-derived consortium with the lowest levels of LCN2. These ‘passaged’ consortia were subsequently gavaged into recently weaned germ-free mice fed the Adult Mirpur diet. Recipients of cecal contents from mice with the high levels of LCN2 had significantly higher levels of serum LCN2 (fig. S4B), C3 (fig. S4C), IL-17A (fig. S4D), and IL-6 (fig. S4E), as well as lower weight gain compared to recipients of clarified cecal contents from mice with low serum LCN2 (fig. S4F, table S7A,B).

We performed full-length 16S rRNA sequencing on DNA extracted from cecal contents of these mice colonized with the intact or ‘passaged’ culture collections from the two groups of women. The cecal bacterial community compositions of mice that received intact culture collections were distinct from the compositions of mice that had received clarified ‘passaged’ cecal contents (fig. S4G). Notably, the absolute abundances of *Ligilactobacillus salivarius, Streptococcus gordonii, Granulicatella adiacens, Streptococcus infantarius, Faecalibaculum rodentium* and *Escherichia ruysiae* were enriched (i.e., higher compared to all other groups) in mice that had been colonized with passaged cecal contents derived from undernourished women which elicited high levels of LCN2 (fig. S4H, table S7C,D). *Escherichia flexneri*, a known enteric pathogen was elevated in mice with systemic inflammation (fig. S4H) and also significantly higher in the duodenal aspirates of women with EED (Fig. 2A, table S10B,C).

In mice colonized with passaged cecal contents derived from well-nourished women that elicited low levels of LCN2, the absolute abundances of *Bifidobacterium pseudolongum* subsp. *globosum* and *Streptococcus anginosus* were significantly higher than all other groups and the absolute abundances of *Weisella cibaria* and *Weisella confusa* were diminished (fig. S4H, table S7D). We named these two ‘passaged’ collections of cultured bacteria the ‘adult small intestinal-low BMI’ (aSI-L) consortium and the ‘adult small intestinal-healthy BMI’ (aSI-H), consortium, respectively. We next compared the effects of these two consortia on mucosal and immune and epithelial responses in adult female mice.

#### Transcriptional responses of small intestinal epithelial cell lineages to EED versus healthy bacterial consortia –

Duodenal, jejunal, and ileal intestinal segments were collected from adult female mice fed the ‘Adult Mirpur’ diet that had been colonized for >5 weeks with the aSI-L or aSI-H consortia (see *‘Tissue collection from adult female mice’* in Methods for how these segments were operationally defined). No statistically significant differences in villus length or crypt depth were observed along the length of the small intestines between animals belonging to the two treatment groups (table S8). We subsequently performed single nucleus RNA-Sequencing (snRNA-Seq) to assess intestinal cellular responses to the aSI-L compared to aSI-H consortium, A total of 45,048, 47,461 and 36,441 nuclei were collected from three intestinal segments (duodenum, jejunum, and ileum; n=4 mice/group, see Methods for quality control). The resulting transcriptomes were categorized into epithelial and mesenchymal cell clusters (lineages) based on marker gene expression (Fig. 3A), with enterocytes making up the largest proportion of nuclei. Enterocytes were organized into subclusters representing subpopulations positioned at different locations along the crypt-villus axis (25, 26).

‘Pseudo-bulk’ analysis of epithelial cell clusters revealed statistically significant elevations in transcripts related to inflammation in aSI-L compared to aSI-H mice (Fig. 3B; see table S8B–D for full lists of differentially expressed genes across cell clusters in each small intestinal segment and along the crypt-to-villus axis). Enterocytes exhibited a greater immunoinflammatory response to the aSI-L compared with aSI-H consortium; for example, genes with significantly increased expression in two or more of the small intestinal segments included those encoding (i) inducible nitric oxide synthase (*Nos2*) which produces the antimicrobial agent nitric oxide, an inflammatory caspase (*Casp4*), and a secreted calcium-dependent phospholipase A2 family member with bactericidal activity (*Pla2g5*) (all three intestinal segments); (ii) dual oxidase 2 (*Duox2*), a nicotinamide adenine dinucleotide phosphate oxidase that is induced to release reactive oxygen species in response to intestinal inflammation(27), the pattern recognition receptor alpha-protein kinase 1 (*Alpk1*) along with its signaling adaptor tumor necrosis factor receptor-associated factor (TRAF)-interacting protein with forkhead-associated domain (*Tifa*), the antimicrobial peptide regenerating islet-derived protein 3 gamma (*Reg3g*), and the antiviral innate immune effector protein apolipoprotein B mRNA-editing enzyme catalytic polypeptide-like 3 (*Apobec3*) (duodenum and jejunum); (iii) glycolytic genes known to be upregulated with inflammation (*Hk2*, *Pfkfb3*) (jejunum and ileum); and (iv) a chemokine involved in homing of lymphocytes to the mucosa (*Ccl28*) (duodenum and ileum). Genes whose expression was significantly increased in more limited regions included: (i) an antimicrobial peptide (regenerating islet-derived protein 3 beta, *Reg3b*) and a chemokine that recruits lymphocytes including Th17 cells (*Ccl20*) in the duodenum; (ii) serum amyloid A, which can promote Th17 responses(28), and a laminin subunit (*Lamb3*) associated with intestinal inflammation(29) in the jejunum and (iii) MHCII complex components (*H2-Aa*, *H2-Ab1*, *H2-Eb1*, *Cd74*) and a cysteine protease implicated in processing peptides for MHCII presentation (*Lgmn*) in the ileum.

Along the length of the small intestine, enterocytes in aSI-L compared to aSI-H adult female mice also exhibited statistically significant elevations in transcripts related to the mucus barrier, including cell-surface presented mucins (*Muc4*, *Muc13*) and mucin-modifying enzymes affecting disulfide bond linkages (*Qsox1*) and transfer of sugar and sulfate moieties (*B3galt5*, *B3gnt7*, *Chst4*, *Fut2*, *Gcnt3*, *St6galnac3*) (Fig. 3B). A number of enterocyte transcripts elevated in the aSI-L group have been previously shown to be upregulated in intestinal epithelial cells in response to Th17-cell induction with segmented filamentous bacteria in conventionally-raised mice (*e.g. Duox2, Fut2, Reg3b*, *Reg3g, Saa1*, *Saa3*)(30) and/or upregulated upon exposure of an intestinal cell line to IL-22 which is produced by Th17 cells (*e.g. B3gnt7*, *Dmbt1*, *Duox2*, *Fut2*, *Gcnt3*, *Hk2*, *Nos2*, *Socs3*, *Tifa*, *Ubd*)(31).

In the aSI-L group, transcripts related to innate immune response pathways were also significantly elevated in goblet and Paneth cells; e.g., *Reg3g*, *Alpk1*, *Tifa*, *Casp4*, *Mmp28*, *Socs3* in goblet cells and *Lgmn*, *Fut2*, and *Socs3* in Paneth cells (table S8B–D). Duodenal goblet cells also exhibited significant reductions in expression of *Mxd1*, a transcriptional repressor that marks fully differentiated goblet cells (32) and *Zg16*, a secreted protein with lectin domains that can aggregate Gram-positive bacteria to limit their penetrance through the mucus layer (33).

Cross-referencing the protein products of genes whose expression was significantly elevated in the duodenum of aSI-L mice with duodenal proteins that were significantly increased in women with EED revealed shared elevations involving metabolic changes associated with inflammation (*Hk2*, *Pfkfb4*), myeloid cell survival and proliferation (*Il34*), and regulation of IL-22/STAT3 signaling (*Ptk6*) (fig. S5A,B).

#### Immune cell profiling and assay of mucosal barrier function –

To assess immune cell representation in the small intestinal lamina propria, we colonized additional groups of germ-free adult female mice fed the Adult Mirpur diet by horizontal transmission (coprophagia) from previously-colonized aSI-L and aSI-H mice (the same groups used for snRNA-Seq analyses). The resulting 16–18-week-old aSI-L mice had significantly elevated serum levels of LCN2 compared to their aSI-H counterparts (Fig. 3C, table S8E). Flow cytometry of lymphocytic and myeloid populations demonstrated significant elevations in the absolute numbers of Th17 cells in the small intestinal lamina propria of aSI-L mice and elevations in the percentages of Th17, γδT, and RORγt^+^ regulatory T cells (Fig. 3D, table S8F,G) – consistent with the Th17-associated enterocyte transcriptional responses described above.

The Th17–IL-22 axis is implicated in both improvements and pathologic disruption of gut barrier function (34). The elevation in serum LCN2 supports a reduction in gut barrier function in the aSI-L group in association with the observed Th17 immune response. Assessment of IL-22 levels in duodenal intestinal homogenates identified a significant increase in the aSI-L group (Fig. 3E). IL-22 reduces ZO-1 expression in intestinal cells (35). Immunostaining of proximal SI enterocytes for the tight junction protein zonula occludens-1 (ZO-1) demonstrated a reduction in aSI-L compared to aSI-H animals (Fig. 3F,G, table S8E). Based on these observations, we directly measured gut permeability by gastric gavage of FITC-4kDa dextran in aSI-L and aSI-H mice. The significant increase in passage of FITC-dextran into the serum of aSI-L mice indicated elevated gut permeability (Fig. 3H, table S8H). Together, these data suggest that the aSI-L consortium imparts a Th17 intestinal mucosal immune response associated with impaired gut barrier integrity in adult female gnotobiotic mice.

### Intergenerational (dam-to-pup) transmission of the aSI-L and aSI-H consortia

To explore the consequences of intergenerational transmission of the EED-associated, inflammatory bacterial consortium (aSI-L), we colonized female and male germ-free mice fed the Adult Mirpur diet with either the aSI-L or aSI-H consortium for one week prior to onset of mating. Their offspring were euthanized on postnatal day 37 (P37) (n=35 offspring/group; Fig. 4A). Offspring of dams harboring the aSI-L consortium exhibited elevated systemic inflammation, as quantified by serum LCN2 (Fig. 4B), IL-17A (Fig. 4C), MMP8 (14) (fig. S6A), and CCL20 (C-C motif ligand 20, also known as Macrophage inflammatory protein-3a) (fig. S6B, table S9A). Post-weaning weight gain was significantly reduced in P37 offspring of dams harboring the human EED donor-derived bacterial consortium. Microcomputed tomography of their femurs revealed that the reduced weight gain in aSI-L mice was accompanied by reduced trabecular bone mineral density and increased cortical bone porosity compared to their aSI-H counterparts (Fig. 4D,E, table S9B,C).

#### Intestinal transcriptional responses in P37 offspring –

We performed bulk RNA-Seq on the duodenum and ileum of a subset of P37 offspring (n=15–16 mice/group). Genes that had significantly higher expression in the duodenums of aSI-L compared to aSI-H offspring included (i) those involved in innate immunity (e.g., *Nos2*, *Duox2* and *Duoxa2*) (fig. S6C,D, table S9D), (ii) *Rorc*, the transcription factor that enables differentiation of Th17 cells (Fig. 4F, table S9D), in agreement with the elevated number of Th17 cells observed in adult aSI-L female mice (Fig. 3D), (iii) components of Reactome pathways involved in antimicrobial responses (‘Alpha-defensins’) and (iv) genes related to DNA damage and repair (‘DNA Repair’, ‘DNA Double Strand Break Response’, ‘DNA Damage/Telomere Stress Induced Senescence’) (table S9E).

Genes whose expression was significantly elevated in the intestines in aSI-L P37 mice that are homologues of protein products enriched in the duodenal mucosal proteomes of women with EED include those involved in myeloid cell survival and proliferation (*Il34, Ly6d*), cellular responses to inflammation (*Fkbp5, Il4i1*), endothelial cell survival and vascular morphogenesis (*Kdr* which encodes VEGFR2, *Cd34*, *Mmp17*) (fig. S7A,B, table S9F).

#### Correlations between bacterial abundances and serum proteins –

We quantified 96 serum proteins (O-link Target 96) in a subset of P37 offspring (n=14 mice/group). Three proteins whose levels were significantly different between P37 aSI-L and aSI-H offspring were also significantly different in the plasma of undernourished compared to healthy women: CXCL1 (diminished in aSI-L and EED, fig. S8A), ENO2 (enolase 2, higher in aSI-L and EED, fig. S8B) and IL-17 (higher in aSI-L and EED, table S9A). We subsequently performed a correlational analysis between bacterial abundances in cecal contents and levels of these proteins. The absolute abundances of *B*. *globosum, Escherichia fergusonii, E. ruysiae*, and *S. infantarius* were negatively correlated with proteins involved in the Th17 axis – CCL20 and IL-17A – and positively correlated with Ectodysplasin A2 receptor (EDA2R, fig. S8C) which is involved in non-canonical NF-kB signaling(36). The absolute abundances of all four of these bacteria were higher in aSI-H offspring. In addition, the absolute abundances of *Eubacterium sp00587695*, *Akkermansia muciniphila*, and *F. rodentium*, which were higher in aSI-L offspring, were positively correlated with CCL20 and IL-17A while negatively correlated with myeloid cell recruiters (C-C motif ligands 2 and 3, CCL2, CCL3), mediators of cell proliferation (TGFa; MIA), and tissue remodelers (TNF Receptor Superfamily Member 12a, EDA2R). Together, these results suggest these organisms are candidate mediators of Th17-driven immune responses and myeloid-driven tissue remodeling in this preclinical model.

### Correlations between duodenal bacteria associated with immunoinflammatory responses in gnotobiotic mice with duodenal mucosal proteins in Bangladeshi women

We matched taxa whose absolute abundances were significantly different between adult aSI-L and aSI-H female mice and/or their P37 offspring (fig. S9A) to taxa in the duodenal aspirates of human donors (fig. S9B). We subsequently correlated the absolute abundances of aSI-L bacterial species that were present in the duodenal aspirates of women with proteins quantified in their duodenal mucosal biopsies (Fig. 4G, table S10A). The absolute abundance of *G. adiacens* in duodenal aspirates from women with EED exhibited the most significant correlations with duodenal mucosal proteins, followed by *S. gordonii* and *Hemophilus parainfluenzae* (Fig. 4H). In women, the absolute abundances of these three bacteria were positively correlated with complement proteins (C3, C1QTNF5, C1QL3, C4A/B, C5/6), cytokines (IL-6R, IL-9, IL-15, IL-17RB, IL-18, IL-20RA, IL-21R, IL-25, IL-26), interferons (IFNA1, IFNA8, IFNGR1, IFNGR2), innate immune system modulators (ACE/ACE2 and HLA-G), pattern recognition receptors (SIGLEC1, SIGLEC6, SIGLEC14), and proteins involved in oxidative stress responses (AKR1B1, AKR1B10) (table S10A, fig. S9C). Conversely, the abundances of these bacteria were significantly negatively correlated with proteins involved in the Reactome signaling pathways for Insulin/IGF, Ras, VEGF, PDGF, FGF and GRH-R (Gonadotropin-releasing hormone receptor) (fig. S9D, table S10A,B). No duodenal mucosal proteins were significantly correlated with the absolute abundances of the two aSI-H taxa that were prevalent enough to perform this analysis (*B. globosum* and *S. anginosus*; FDR-corrected Spearman correlations). Together, these data indicate that duodenal bacteria from undernourished women with EED induce pro-inflammatory immune responses in gnotobiotic dams that are transmitted to their offspring, with effects on intestinal barrier function and immune signaling.

### Duodenal bacteria and mucosal proteins that characterize EED in adult Bangladeshi women and children

We compared the duodenal mucosal proteomes of women and children with EED, first seeking proteins that were consistently elevated in adult women and children with histopathologic evidence of severe EED (histopathology score=3) compared to those with normal histology (Fig. 5A, fig. S10). These 159 proteins included: Interleukin-18 receptor 1 (IL18R1), Neutrophil-activating peptide 2 (PPBP), Lysozyme C (LYZ), Toll-like receptor 4 (TLR4), Eotaxin (CCL11), Immunoglobulin A (IGHA1/2), Endothelial monocyte-activating polypeptide 2 (AIMP1), C-C motif chemokines 25 and 28 (CCL25/28) and Complement component 1Q binding protein (C1QBP) (table S10C). Furthermore, this shared duodenal mucosal proteomic signature of EED in women and children included four of the 84 putative biomarkers of EED additionally elevated in plasma of women with EED: S100A9, Hexokinase 1 (HK1), Lectin, Mannose-binding 2 (LMAN2) and Tumor Necrosis Factor Ligand Superfamily Member 14 (TNFRSF14) (see section ‘*Plasma and duodenal proteomes of undernourished women with EED*’, table S1D).

Next, we searched the duodenal mucosa proteomics data from adult women for a set of 247 proteins that were previously found to be negatively correlated with linear growth (LAZ) in children (14). Of these proteins that were negatively correlated with LAZ in duodenal biopsies of children, 34 were significantly higher in duodenal biopsies of women with EED compared to women without pathology (Fig. 5B, table S10D). These 45 proteins included 3 of the 84 putative biomarkers of EED described above: UDP-glucose 4-epimerase (GALE), Dickkopf-like protein 1 (DKKL1) and LMAN2 (Fig. 5C). Collectively, these data highlight the overlapping duodenal mucosal proteomic signatures of EED in women and children as well as those reflected in plasma that may be assessed non-invasively.

Bacteria cultured from children with EED that were associated with healthy offspring growth and reduced inflammation in our mouse model (the ‘child small intestinal non-inflammatory’ consortium, cSI-N (16)) included *P. melanogenica, S. anginosus*, and *Streptococcus cristatus* – these species trended towards increased absolute abundance in the aspirates of women lacking histopathologic evidence of EED (table S2C) – as well as *Streptococcus oralis*, whose abundance was negatively associated with histopathology score (Fig. 2A, table 2B). Moreover, the absolute abundances of *S. cristatus, S. anginosus*, and *P. melanogenica* in duodenal aspirates of adult women were significantly negatively correlated with proteins involved in innate immune signaling pathways (i.e., when the abundances of these bacteria are higher, expression of these innate immune signaling pathways decreases, table S10F,G).

Duodenal bacteria from children with EED that were associated with inflammation in P37 offspring in our previously published intergenerational mouse model (gnotobiotic dam-to-pup transmission, cSI-I (16), table S10E) and were present in aspirates of adult women included *Streptococcus constellatus, Veillonella atypica, Actinomyces naeslundii, H. parainfluenzae*, and *S. gordonii*. In adult women, the absolute abundance of *S. gordonii* increased with severity of EED (Fig. 2A). Furthermore, the absolute abundances of these five taxa were significantly positively correlated with levels of proteins in the duodenal mucosa that comprised the Reactome pathways for ‘Interleukin-12 signaling’, ‘Detoxification of Reactive Oxygen Species’ and ‘GPCR ligand binding’ (GSEA *q*-value<0.05, table S10F,G).

Together, these results suggest the presence of conserved strains within the small intestinal microbiota that contribute to shared pathogenic pathways underlying EED in both adult Bangladeshi women and children living in the same urban locale. These strains were transmitted from dams to pups in our intergenerational gnotobiotic mouse models where they contributed to pathology in offspring. Whether these bacterial strains are vertically transmitted in humans remains to be investigated.

## DISCUSSION

While enumeration of bacteria in the distal gut microbiome through analysis of fecal samples has been the standard approach in most studies of childhood undernutrition (11, 37–40), the small intestine is the primary site of EED. Our previous work established that bacteria in duodenal aspirates from stunted children with EED correlated with markers of enteropathy and growth impairment (14). The current study extends this work to adult Bangladeshi women of reproductive age and focuses on expressed proteins rather than mRNAs (41, 42). Comparing bacterial associations with the duodenal mucosal and plasma proteomes in undernourished women with and without histopathologic evidence of EED we identified a set of plasma proteins that report features of the immunoinflammatory responses observed in the duodenal mucosa of the women with EED. We determined whether discordant features of their microbiota were causally related to enteropathy by using gnotobiotic mice colonized with bacterial consortia cultured from the two groups of women. Proteomic, snRNA-Seq, and flow cytometric analyses demonstrated that the EED donor-derived consortium induced intestinal and serum immunoinflammatory responses in recipient adult gnotobiotic female mice. Moreover, dam-to-pup transmission of the EED-donor derived consortium produced enteropathy in offspring, accompanied by significant reductions in weight gain. Bacterial species whose abundances were elevated in the offspring of dams harboring the EED donor-derived consortium were also significantly correlated with duodenal mucosal proteins that were discriminatory for EED in these cohorts of Bangladeshi women. Intergenerational transmission of these phenotypes supports the hypothesis that vertical microbiota transfer is a mechanism that contributes to perpetuating the cycle of undernutrition and EED.

### Study limitations –

While longitudinal sampling in clinical trials is the gold standard, the invasive nature of esophagogastroduodenoscopy precluded our ability to obtain multiple mucosal biopsies and luminal aspirates along the length of the small intestine. Future clinical trials using capsule-based technology (43) could allow such biogeographic sampling over time. In addition, decompartmentalization of oral bacteria to the small intestinal microbiota (16, 39, 44) and their subsequent contribution to pathophysiology of EED warrant further clinical investigation. Future studies comparing the oral and small intestinal microbiota of undernourished women with EED and their well-nourished counterparts without evidence of enteropathy prior to, during, and after pregnancy could provide insights into the dynamics of these microbial ecosystems, their relationships to maternal health and impairments in pre- and postnatal development of their offspring, and hypotheses about microbial as well as host targets for therapeutic interventions – hypotheses that can be tested in the type of gnotobiotic models described in this report (also see ref. (16)). While the plasma biomarkers of EED in adult women could provide improved, non-invasive ways for diagnosing enteropathy in women prior to pregnancy, their utility should be validated in additional cohorts using cost-effective methods.

In summary, our study provides evidence supporting the notion that the small intestinal microbiota of adult women is a driver of undernutrition with consequences for child development. The results suggest that targeting maternal gut health via the microbiota may be one way to break the intergenerational cycle of undernutrition.

## MATERIALS AND METHODS

### Study Design

This study enrolled undernourished Bangladeshi women of child-bearing age who failed a standard nutritional intervention and their well-nourished counterparts so that esophagogastroduodenoscopy (EGD) could be performed with collection of duodenal aspirates and duodenal mucosal biopsies. Blood and fecal samples were collected concurrently. Bacteria were cultured from the duodenal aspirates of both undernourished women and well-nourished controls to evaluate their effects in gnotobiotic adult female mice and their offspring.

### HUMAN STUDIES

#### Ethics and Inclusion –

This work was performed as part of a long-standing collaboration investigating the role of the gut microbiota in undernutrition that is covered by a memorandum of understanding, between teams led by Tahmeed Ahmed (International Centre for Diarrhoeal Disease Research, (icddr,b) Bangladesh) and Jeffrey Gordon (Washington University in St Louis, USA). Bacterial isolates used in this study were obtained from duodenal aspirates collected from the previously reported Bangladesh EED study, which was approved by the Ethical Review Committee (ERC) at the icddr,b (protocol no: PR-16007; ClinicalTrials.gov number, NCT02812615). Written, informed consent was obtained from all participants. Human biospecimens were transferred to Washington University under a Materials Transfer Agreement (MTA). Bacterial isolates cultured and used in this study are the property of icddr,b and are available under MTA upon request to T.A. and J.I.G.

#### Design and histopathologic scoring of duodenal mucosal biopsies –

All biospecimens from the women in described in this report were collected with approval by the Ethical Review Committee at icddr,b. All participants provided informed, written consent. Details of study design, recruitment, and exclusion criteria for the clinical trials included in this study have been reported elsewhere (15, 17, 18). Briefly, undernourished (BMI < 18.5 kg/m^2^) women 18–45 years old were recruited to the Bangladeshi EED (BEED) study for nutritional therapy. Exclusion criteria included severe anemia (Hb < 8 g/dL), chronic or acute diseases, pregnancy, lactation, drug abuse, known psychiatric disorders, and conditions causing secondary malnutrition. Women whose BMI failed to improve (<10% BMI increase) after three months underwent EGD for collection of duodenal biopsies and dry aspirates. Fecal samples and plasma were collected concurrently.

As part of the BEED study, well-nourished (BMI = 18.5–30 kg/m^2^) women 18–45 years old seeking treatment for dyspepsia who were residing in the same urban slum (Mirpur) in Dhaka, Bangladesh underwent EGD. Those who had normal endoscopic findings were included as healthy controls (15, 17), and duodenal biopsies and aspirates were collected. In the follow-up ‘Normal BMI’ cohort (18), adult women from non-slum sections of Dhaka were included if they were 18–45 years of age and had BMI 20–25 kg/m^2^; duodenal biopsies and aspirates, plasma, and fecal specimens were collected.

Biopsy samples were fixed in 10% buffered formalin solution, and paraffin sections were prepared with hematoxylin and eosin (H&E) staining. Histological scoring criteria are described in detail in ref. (17). Intestinal morphometric parameters measured included: villous atrophy, crypt hyperplasia, and inflammatory infiltrates in the lamina propria. Histological parameters were evaluated as follows: (i) villous atrophy – mild = mild reduction in villous height, subtotal = partial blunting, total = complete atrophy or flattening of villi; (ii) crypt hyperplasia – absent = no crypt elongation, present = crypt elongation or hyperplasia evident; (iii) inflammatory infiltrates – mild = mild increase of inflammatory infiltrates, moderate = lymphoid aggregates present, marked = diffuse and intense inflammatory infiltrates. These descriptive morphometric features were aggregated into histological scores from zero to three, with three indicating the most severe evidence of EED.

### Plasma and duodenal mucosal proteomics

#### Plasma –

Blood (4.5 mL) was collected from undernourished (n=22) and normal-BMI women (n=25) in EDTA-coated tubes and was centrifuged at 3000 × *g* for 10 minutes (20 °C) to separate plasma, which was subsequently aliquoted and stored at −80 °C. A 55 μL aliquot of plasma was used to measure 9,609 unique proteins (SomaScan 11K Assay; SomaLogic).

#### Duodenal and plasma proteomic analyses –

Duodenal biopsies were stored at −80 °C until extraction, at which time they were transferred to ice-cold Tissue Protein Extraction Reagent (T-PER, ThermoFisher 78510) supplemented with protease inhibitor (Roche cOmplete Ultra Protease inhibitor, 481761). Proteins were extracted with Cytiva sample grinding kits (80-6483-37) according to the manufacturer’s recommendations. Briefly, grinding tubes were centrifuged for 1 minute at 18,000 × *g* and excess liquid was removed. 200 μL of ice-cold T-PER containing protease inhibitor was aliquoted into each tube, which was maintained on wet ice. Biopsy samples were transferred to the tissue grinding tubes with sterilized fine point tweezers. Tissue was homogenized manually using the pestles provided in the Cytiva kit until no visible pieces of tissue remained. Homogenized samples were centrifuged at 18,000 × *g* at 4 °C for 20 minutes. The supernatant was transferred to a new 2 mL screw cap tube (Axygen). Tissue homogenates were diluted 1:100 into 1X PBS containing protease inhibitor (Roche cOmplete Ultra) and total protein was quantified using the Micro BCA Protein Assay kit (ThermoFisher 23235). Samples were normalized to 200 μg/mL in 1X PBS and stored at −80 °C. The SomaScan 7K Assay (SomaLogic) was used to measure 6,401 unique SOMAmers (which bind specific proteins and serve as a proxy for the abundance of the corresponding protein) in duodenal biopsy homogenates.

Output from SomaScan proteomic assays were quality-checked and normalized according to the manufacturer’s recommendations using hybridization controls, followed by median signal normalization across pooled calibrator replicates within the run. Median signal normalization was performed using Adaptive Normalization by Maximum Likelihood (ANML). ANML-normalized data were log_10_-transformed. Any SOMAmers flagged as not passing pooled QC thresholds for determining the accuracy of median replicate signal compared to a reference were removed.

Plasma and duodenal mucosal proteins with the lowest 10% expression were removed, resulting in 9,448 plasma and 6,626 duodenal tissue aptamers tested. Differential abundance analysis was performed using the R package *limma* (v3.60.6 (45)). A linear model was used to fit each aptamer using *lmFit* with a design matrix specifying cohort as the coefficient for plasma samples and histopathology score as the coefficient to be estimated for duodenal biopsies. Empirical Bayes moderation of variance (46) was applied with *eBayes* and Holm-Bonferroni multiple hypothesis correction was included with *topTable(p.adjust = ‘holm’)*.

Proteins identified in plasma samples were tested for their association with BMI using the Significance Analysis of Microarrays (SAM (47)). The same filtering criteria were applied to the plasma SomaScan data as for the differential protein abundance analysis. Using the R package *SAM* (built on *samr*, v3.0), a d-statistic was computed for ‘Quantitative’ response type, standard regression method, 10 k-nearest neighbors and 1000 permutations. The 90^th^ percentile FDR < 0.05 was used to select the delta value d=1.08; features with *q*-value < 0.05 were considered significant.

Enrichment analysis of plasma and duodenal mucosal proteins was performed using *clusterProfiler* (48) (v4.12.6). The association of plasma aptamers with BMI (d-score) and duodenal mucosal aptamer differences by histopathology score (*limma* logFC) were used to rank proteins. Gene Ontology (GO) Biological Process, Molecular Function, and Cellular Component databases were queried with *gseGO(geneList, orgDb=org.Hs.eg.db, pAdjustMethod = ‘BH’, minGSSize=20, pvalueCutoff=0.05)*. Reactome pathway enrichment was analyzed with *gsePathway(geneList, pvalueCutoff = 0.1, organism= ‘human’, pAdjustMethod = ‘BH’, by=‘fgsea’). P*-values were adjusted using the Benjamini-Hochberg method.

### Sequencing full-length 16S rRNA amplicons generated from duodenal samples

#### DNA isolation from duodenal aspirates –

Duodenal aspirates were thawed on wet ice anaerobically for concurrent culturing (see *‘Culturing bacteria from duodenal aspirates’*) and low biomass DNA extraction with the Arcturus PicoPure DNA Extraction Kit (KIT0103, Applied Biosystems). For duodenal aspirates obtained from the BEED study, a spike-in consisting of 4.95 × 10^5^ cells of *Alicyclobacillus acidophilus* (49) was added. For duodenal aspirates obtained from the second healthy cohort, a premixed *Allobacillus halotolerans* and *Imtechella halotolerans* spike-in standard (ZymoBIOMICS D6320–10) was diluted in autoclaved 1X PBS to the same number of cells added to the samples from the BEED study (4.95 × 10^5^ of each strain) in a final volume of 30 μL. 50 μL of duodenal aspirate was added to the spike-in. Aspirates were centrifuged at 5,000 × *g* for 10 minutes at 10 °C. During centrifugation, lyophilized Proteinase K was centrifuged briefly prior to resuspension in 155 μL Reconstitution Buffer, then maintained on wet ice. After centrifugation of the aspirates, 50 μL of the supernatant was removed and 50 μL of reconstituted Proteinase K was added to the cell pellet with gentle pipetting to mix. Samples were incubated at 65 °C for 10 hours then 95 °C for 10 minutes in a digital oven (Quincy Lab, Inc Model 30E), prior to overnight freezing at −20 °C. 250 μL of 0.1 mm zirconia silica beads and one 3.97 mm steel ball were used to bead beat samples in a Biopsec Minibeadbeater-96 for 4 minutes in 500 μL 2X buffer A (0.2 M NaCL, 0.2 M Tris, 0.02 M EDTA), 210 μL 20% SDS, and 510 μL phenol:chloroform:isoamyl-alcohol (pH 8). Samples were centrifuged at 3,200 × *g* for 4 minutes at 4 °C; 180 μL of the resulting aqueous phase extract was then added to a 720 μL 675:45 mixture of Qiagen Buffer PM and 3 M NaOAc (pH 5.5), mixed, and subsequently transferred to a Qiagen QiaQuick Gel Extraction column. The remaining DNA purification steps were performed with Qiagen Buffer PE and Buffer EB according to the manufacturer’s specifications.

#### Library preparation, sequencing, and analyses –

DNA concentration was quantified with Qubit. Kinnex full-length 16S rRNA libraries were prepared according to the manufacturer’s specifications. De-concatenation was performed using Read Segmentation and resulting demultiplexed reads were trimmed (TrimGalore v0.6.6 –quality 20). Adaptors were removed from trimmed reads with Cutadapt (-g AGRGTTYGATYMTGGCTCAG… AAGTCGTAACAAGGTARCY --error-rate 0.1 –revcomp). Subsequent processing of 16S rRNA reads was performed with the *dada2* (50) pipeline with minor modifications to the *filterAndTrim* step *(minLen=1000, maxLen=1600, maxN=0, rm.phix=TRUE)*. Error rate (*learnErrors*) learning was performed for each fastq file; dereplication (*deRepFastq*) and dada steps were performed with standard parameters. Taxonomic predictions were generated with *assignTaxonomy* using the GreenGenes (2024) database.

In *phyloseq* (51) (v1.48.0), bacterial reads from duodenal aspirates were normalized to spike-in counts to approximate absolute abundance in cecal contents. For all analyses, ASV abundances were collapsed into species-level abundances using *tax_glom(taxrank = “Species”)*. Bacterial species with a prevalence of <50% of the smallest group of participants involved in each comparison were removed. In total, 21,493 unique ASVs and 887 species were identified in duodenal aspirate samples; 127 species were present in at least 10 samples. Statistical analysis of differences in the absolute abundances of bacterial taxa was performed using a zero-inflated Gaussian mixture model with *metagenomeSeq* (52). Briefly, counts were normalized using cumulative-sum scaling (*cumNormStatFast*). The statistical model *(~histopathology_score + extraction_batch*) was used to determine significant associations with histopathology score as a numeric with *fitZig*. Linear models comparing bacterial abundances in duodenal aspirates were performed on log_10_-transformed abundances treating histopathology score as a factor *(histopathology_score_group + extraction_batch* and *~disease_status + extraction_batch*).

### Associating duodenal bacteria with duodenal mucosal proteins

To identify bacteria with abundances that were associated with host protein levels independent of EED status, we fit a main effects linear model for each bacterium–protein pair using the R package *limma* with the design formula: *aptamer ~ bacterial_abundance + EED_status*, where *aptamer* represents log_10_-transformed SomaScan values that were filtered to remove the 30% of aptamers with the lowest variance, *bacterial_abundance* represents log_10_-transformed absolute abundances in duodenal aspirates or relative abundances in fecal samples, and *EED_status* is a binary indicator of disease status (histopathology score ≥ 1 or histopathology score = 0). For each of the bacterial taxa, all protein targets were fit with the linear model using *lmFit*, and the ß_1_ coefficient was extracted (slope). Empirical Bayes moderation of residual variances was implemented with *eBayes(robust = TRUE)*. Multiple hypotheses were corrected for using the Benjamini-Hochberg false discovery rate correction across all bacterium-protein pairs. Associations with *q*<0.05 were considered significant.

To identify bacteria-protein pairs whose linear relationship differed significantly between women with and without EED (53, 54), we used the following design formula: *aptamer ~ bacterial_abundance + EED_status + bacterial_abundance:EED_status*. This model yielded the main effect of bacterial abundance in healthy women (ß_1_), the effect of EED status (ß_2_) and the interaction term (ß_3_) representing the differences in slopes between bacterial and protein abundance. As above, empirical Bayes moderation and multiple hypothesis correction were applied. Enrichment analyses on proteins that were significantly associated with bacterial abundance in this disease-specific model were performed using the R package *gProfiler2* (55) with the function *gost(query, organism = “hsapiens”, correction_method = “fdr”, significant = TRUE, evcodes = FALSE, mutli_query = FALSE)*. Proteins that were significantly linearly related to the abundance of bacteria either positively or negatively were queried separately.

### GNOTOBIOTIC MOUSE EXPERIMENTS

All experiments involving mice were performed using protocols approved by Washington University Animal Studies Committee. Mice were housed in plastic flexible film gnotobiotic isolators (Class Biologically Clean Ltd., Madison, WI) at 23 °C under a strict 12-hour light cycle (lights on at 0700h). Autoclaved paper ‘shepherd shacks’ were kept in each cage to facilitate the natural nesting behaviors and for environmental enrichment.

#### Diets –

The ‘Adult Mirpur’ diet was designed based on 24-hour dietary recall surveys and food frequency questionnaires taken from adults living in the Mirpur district of Dhaka, Bangladesh who were enrolled in the BEED study. A pelleted, sterile version of this diet was manufactured by Dyets, Inc. (Bethlehem, PA). The quantity of each ingredient used to prepare the diet is provided in ref. (16). The composition of the ‘Mirpur-18’ diet was based on Bangladeshi complementary feeding practices for 18-month-old children living in Mirpur, as defined by quantitative 24-hour dietary recall surveys conducted in the MAL-ED study (56). This diet was manufactured according to previously described protocol (14).

The sterility of irradiated diets was confirmed by culture in (i) BHI broth, (ii) Nutrient broth, and (iii) Sabouraud-dextran broth (all from Difco) for one week at 37 °C under aerobic conditions, and in reduced Tryptic Soy broth (Difco) supplemented with 0.05% L-cysteine HCl under anaerobic conditions. All diets were stored at −20 °C prior to use. Nutritional analysis of each irradiated diet was conducted by Nestlé Purina Analytical Laboratories previously (St. Louis, MO), see ref. (16).

### snRNA-Seq of duodenum, jejunum and ileum

#### Isolation of nuclei and library preparation –

Intestinal segments from the duodenum, jejunum, and terminal ileum (see *‘Tissue collection from adult female mice’*) were processed as previously described (57). Tissues were minced in a buffer of 25 mM citric acid, 0.25 M sucrose, 0.1% NP-40, and 1X protease inhibitor (Roche); sheared using a Dounce homogenizer (Wheaton) to release nuclei; washed 3 times with a buffer of 25 mM citric acid, 0.25 M sucrose, and 1X protease inhibitor (Roche cOmplete Ultra); filtered through 100 μm-, 40 μm-, 20 μm-, and 5 μm-diameter strainers, and resuspended in a buffer consisting of 5 mM KCl, 3 mM MgCl_2_, 50 mM Tris, 1 mM DTT, 0.4 U/μL RNase inhibitor (Sigma), plus 0.4 U/μL Superase inhibitor (ThermoFisher Scientific). The Chromium Next GEM 3’ kit v3.1 (10x Genomics) was used to generate gel bead-in-emulsions then cDNA libraries using 7000 nuclei/sample. After quality assessment with a BioAnalyzer, balanced libraries were sequenced on an Illumina NovaSeq 6000 instrument [150 nt paired-end reads; 2.74 × 10^8^ ± 3.35 × 10^7^ reads/sample (mean ± SD)].

#### Data analysis –

Sequencing reads were demultiplexed and aligned to the mouse reference genome (GRCm38/mm10) with *CellRanger* 5.0 (10x Genomics) and denoised using *CellBender* (58). Sample datasets were further processed using the Seurat v4 framework (59) to remove low quality nuclei (< 400 UMI, < 200 or > 8000 genes, > 5% mitochondrial reads, and/or > 60% ribosomal reads), normalize counts (*SCTransform*), remove predicted doublets (*DoubletFinder*), integrate samples within each tissue type (duodenum, jejunum, or ileum) using anchors identified by reverse PCA, and to cluster nuclei using *FindNeighbors* (dimensions 1:30) and *FindClusters* (resolution 1.4). Cell types were annotated by matching cluster gene markers (*FindMarkers*) with reported cell-type markers (25, 26, 60). ‘Pseudobulk’ analysis was performed by aggregating counts within each cell type designation for clusters with > 100 nuclei/group, then performing comparisons across treatment groups with *DESeq2*. Gene set enrichment analysis (GSEA) was performed with *fgsea*. Human homologues to the mouse genes that were differentially expressed in the duodenums of aSI-L and aSI-H adult female mice were identified using the R package *homologene* with the function *human2mouse(db = homologeneData)*.

### Quantitation of gut bacteria

#### DNA extraction –

The absolute abundances of bacterial species were determined using methods described above with minor modifications. In brief, ‘spike-in’ bacterial strains whose genomes are easily differentiated from those of gut bacteria were added to each weighed frozen sample of intestinal contents prior to DNA isolation and preparation of barcoded libraries. For initial tests of the pooled culture collections and clarified cecal contents from mice harboring high and low levels of LCN2, *Alicyclobacillus acidiphilus* spike-in was added to frozen cecal contents after weighing (1.33 × 10^7^ cells per aliquot of cecal contents; DSM 14558; GenBank assembly accession: GCA_001544355.1). For experiments involving adult female mice and P37 aSI-L and aSI-H animals, a commercial 1:1 mix of *Imtechella halotolerans* and *Allobacillus halotolerans* was added (1.33 × 10^7^ cells per aliquot of cecal contents; ZymoBIOMICS product number D6320–10).

DNA was extracted from flash-frozen cecal contents obtained stored at −80 °C. To obtain crude DNA, frozen cecal contents (~50 mg) were subjected to 4 minutes of bead-beating in a 2 mL tube containing 0.1 mm silica beads, one 3.97 mm steel ball, 500 μL phenol:chloroform:isoamyl alcohol and 710 μL of a 500:210 (v/v) mixture of 2X buffer A:20%SDS. Crude DNA was subsequently purified (QIAquick 96 PCR Purification Kit) and quantified (Qubit). The final DNA fragment size distribution was determined using an Agilent Technologies 4200 TapeStation. Purified DNA samples were normalized to 0.4 ng/μL prior to preparation of Kinnex PacBio full-length 16S rRNA amplicon libraries, as described above in ‘*Full-length 16S rRNA sequencing of human samples*’.

#### Differential abundance analyses –

Raw PacBio HiFi reads were processed as described above in *Library preparation, sequencing, and analyses*. The resulting ASVs were collapsed to unique species using *tax_glom(taxrank = “Species”)*. Species counts were normalized to spike-in counts to approximate absolute abundance, as recommended by the manufacturer. Bacterial reads from mouse samples were aligned to the 16S rRNA sequences from the human datasets. Bacteria with <98.7% nucleotide sequence identity to a strain identified in humans were removed prior to statistical analyses, as well as bacteria that were detectable in fewer than 3 mice. The statistical significance of observed differences in the absolute abundances between groups was tested using a non-parametric Wilcoxon rank-sum test with FDR correction (Benjamini-Hochberg) on log_10_-transformed absolute abundances.

#### Matching taxa in mice with human samples –

Best matches between bacterial taxa that colonized mice and their human donors were identified using two parallel approaches. For unique species present in gnotobiotic mice and duodenal aspirates or fecal samples, sequences were converted to *DNAStringSet* objects using the package *Biostrings* (v2.72.1; https://github.com/Bioconductor/Biostrings). First, a k-mer based approach was used to find the best matches between all mouse bacterial 16S rRNA sequences and all human duodenal aspirate or fecal bacterial 16S rRNA sequences using the package *stringdist* (v0.9.15) as follows: *stringdist(seq, ref_seq, method= ‘qgram’, q=12)*, the pairwise identities for the best matches were calculated with *pairwiseAlignment(seq1, seq2, type=‘global’)*. Second, full alignments were used to compare the sequences of species-level representatives (from *tax_glom(taxrank=“Species”)*) that colonize mice with the sequences of species-level representatives in duodenal aspirates and fecal samples, again using *pairwiseAlignment*.

#### Correlations between taxa associated with pathology in mice and duodenal mucosal proteins in adult women –

Taxa that were associated with pathology in P37 offspring were identified in the duodenal aspirates of women using the methods described above in ‘*Matching taxa in mice with human samples*’. Correlations between the abundances of these taxa and levels of duodenal mucosal proteins in matched samples were calculated using log_10_-transformed bacterial abundances and SomaScan data normalized as described above in ‘*Block sparse partial least-squares discriminant analysis*’. Spearman correlations [*cor.test(bacterial_abundance, protein_abundance, method=‘spearman’)*] were calculated for every possible bacterial-protein pair; *P*-values were adjusted using the Benjamini-Hochberg correction. Enrichment analyses were performed using the R package *gprofiler2* (55) with the function *gost(query, organism = “hsapiens”, correction_method = “fdr”, significant = TRUE, evcodes = FALSE, mutli_query = FALSE)*, where the set of proteins that were significantly positively and negatively correlated with each bacterium were queried separately.

### Statistical Analysis

Statistical methods applied to DNA and RNA sequencing as well as proteomic datasets are detailed in the Methods sections describing how these datasets were generated (also see ‘Materials and Methods’ in Supplementary Materials). Non-parametric (i.e., Wilcoxon rank-sum) tests were routinely used to determine differences in bacterial abundance and host metrics in gnotobiotic mouse experiments. Where applicable, the Benjamini-Hochberg FDR correction was applied.

## Supplementary Material

Supplement 1 **Table S1. Biospecimens acquired and proteomics analysis of clinical cohorts. (A)** Metadata and biospecimens collected from human studies. **(B)** (i) Duodenal mucosal proteins that differed significantly between women with EED (histopathology score ≥ 1) and without EED (histopathology score 0) (*limma* with empirical Bayes moderation). (ii) Reactome pathways that were significantly enriched in the duodenal mucosal proteome of women with EED compared to those without histologic evidence of EED (from i, gene-set enrichment analysis performed with *clusterProfiler*). **(C)** (i) Plasma proteins that differed significantly between the Low BMI and Normal BMI cohorts (*limma* with empirical Bayes moderation). (ii) Plasma proteins that were significantly associated with BMI (Significance Analysis of Microarrays, SAM). **(D)** Proteins that were both negatively associated with BMI in plasma (as in C(ii)) and higher in the duodenal biopsies of women with histopathologic evidence of EED (as in B(i)).

Supplement 2 **Table S2. Duodenal microbiota of women with EED. (A)** Absolute abundances of bacterial taxa in duodenal aspirates (log_10_-transformed 16S rRNA counts per gram aspirate). (i) Present in at least n=10/80 duodenal aspirate samples. (ii) All bacteria detected. **(B)** Results of zero-inflated Gaussian mixture model testing for significant associations between the absolute abundance of bacterial taxa in duodenal aspirates and histopathology score. **(C)** Duodenal bacterial taxa whose absolute abundance differed between (i) histopathology scores and (ii) women with (histopathology score ≥ 1) or without (histopathology score 0) evidence of EED (linear models on log_10_-transformed absolute abundances).

Supplement 3 **Table S3. Analyses associating duodenal bacterial taxa and duodenal mucosal proteins in women. (A,B)** Duodenal mucosal proteins (A) and bacteria in duodenal aspirates (B) that were predictive of EED status (histopathology score ≥ 1 vs. 0 (Data Integration Analysis for Biomarker discovery using Latent variable approaches for Omics studies, ‘DIABLO’). **(C)** Results of linear models relating absolute abundances of taxa in duodenal aspirates to duodenal mucosal protein levels. **(D,E)** Functional enrichment analysis performed on duodenal mucosal proteins that were positively (D) or negatively (E) related to absolute abundance of bacterial taxa (*gProfiler2* over-representation test).

Supplement 4 **Table S4. Fecal microbiota of women in the Low BMI and Normal BMI cohorts. (A)** Relative abundance (percent) of bacterial taxa in fecal samples. (i) Present in at least n=12/63 fecal samples. (ii) All bacteria detected. **(B)** Statistical tests for differential abundance of bacteria in fecal samples obtained from women in the two cohorts (Benjamini Hochberg-adjusted Wilcoxon rank-sum tests).

Supplement 5 **Table S7. Quantification of protein biomarkers and bacterial abundance in mice colonized with culture collections and clarified cecal contents. (A)** Quantification of serum biomarkers in (i) mice colonized with pooled culture collections from Low BMI and Well-nourished women and (ii) mice colonized with cecal contents obtained from mice harboring intact culture collections from Well-nourished (NBMI) or Low BMI (LBMI) donors that produced high (Hi) or low (Lo) levels of LCN2 (see fig. S4). **(B)** Changes in body mass over time in mice described in A(ii). **(C,D)** Quantitation of bacterial taxa (log_10_-transformed 16S rRNA counts per g cecal contents) (C) and statistical tests of differences in absolute abundance (D) from mice in A(i) (Benjamini Hochberg-adjusted Wilcoxon rank-sum tests) and A(ii) (Benjamini Hochbergadjusted Kruskal Wallis tests with Tukey’s post-hoc).

Supplement 6 **Table S8. Small intestinal histomorphometric and snRNA-Seq plus proteomic analyses of adult female aSI-L and aSI-H mice. (A)** Histomorphometric analysis (10 measurements/small intestinal segment from n=4–5 mice/treatment group). Statistical significance assessed by linear mixed-effects models (*Measure ~ Microbiota + (1 | Mouse_ID*)). **(B-D)** ‘Pseudo-bulk’ differential expression analysis of snRNA-Seq data acquired from the duodenum (B), jejunum (C), and ileum (D) of adult female aSI-L mice (positive log_2_-fold change) and aSI-H mice (negative log_2_-fold change) (Benjamini Hochberg-adjusted Wald test using *DESeq2*). **(E)** Quantification of protein biomarkers in serum (i, ELISA) and intestinal tissue (i, Luminex and ii, immunofluorescence) of aSI-L and aSI-H adult female mice. **(F)** Immune cells quantified by flow cytometry in the small intestinal and colonic lamina propria of aSI-L and aSI-H adult females. **(G)** Antibodies used for flow cytometry in F. **(H)** Quantification of FITC-conjugated 4 kDa dextran in the serum of aSI-L and aSI-H adult females.

Supplement 7 **Table S9. Serum proteomic, bone micro-computed tomography and intestinal bulk RNA-Seq analyses of P37 offspring of aSI-L and aSI-H dams. (A)** Serum proteins quantified with ELISAs (i) and the Olink Target 96 Mouse Exploratory platform (ii, Benjamini Hochberg-adjusted Wilcoxon rank-sum tests). **(B)** Micro-computed tomography measurements of femurs: (i), cortical bone tissue; (ii), trabecular bone tissue (two-sample two-tailed t-tests). **(C)** Body weights. **(D)** Differential gene expression between aSI-L offspring (positive log_2_-fold change) and aSI-H offspring (negative log_2_-fold change) (Benjamini Hochberg-adjusted Wald tests using *DESeq2*, conducted separately for each tissue). **(E)** Results of GSEA performed on differentially expressed genes in intestinal tissue (as in D). **(F)** Differential expression of genes encoding mouse homologs of duodenal mucosal proteins that were significantly higher in women with EED (as in Table S1B). (i), Mouse homologs of proteins that were higher in women with EED and more highly expressed in the intestinal tissue of aSI-L offspring. (ii), Mouse homologs to proteins that were diminished in women with EED compared to healthy women and more highly expressed in the intestinal tissue of aSI-H offspring.

Supplement 8 **Table S10. Correlations between bacterial taxa associated with inflammation in pre-clinical mouse models and duodenal mucosal proteins in women. (A)** Significant correlations between the absolute abundances of aSI-L bacteria in duodenal aspirates and levels of proteins quantified in duodenal mucosal biopsies (Spearman correlations were performed for bacteria that were present in greater than 10 women). **(B)** Functional enrichment analysis (*gProfiler2* over-representation test) performed on duodenal mucosal proteins that were significantly correlated with the absolute abundance of aSI-L bacterial taxa in aspirates (from A). **(C)** Duodenal mucosal proteins whose levels were significantly different between women and children with severe EED (histopathology score 3) compared to women and children without pathology (score 0). **(D)** Duodenal mucosal proteins that were significantly negatively correlated with LAZ in children with EED and whose levels were significantly different in the biopsies of adult women with (histopathology score ≥ 1) and without EED (histopathology score 0). **(E)** Full-length 16S rRNA sequencing was performed on DNA extracted from cecal contents of P37 offspring of dams harboring consortia of bacteria cultured from duodenal aspirates obtained from children with EED (16). Sequence and taxonomic matches between these bacteria cultured from children with EED and bacteria detectable in the duodenal aspirates of adult women in the current study are shown. **(F)** Significant correlations between bacteria cultured from children with EED detected in aspirates of women and their duodenal mucosal proteomes (FDR-adjusted Spearman correlations). (i), Bacterial taxa that induced immuno-inflammatory responses in P37 offspring (‘cSI-I’; ref^16^); (ii), Bacterial taxa that did not induce inflammation (‘cSI-N’; ref. (16)). **(G)** GSEA performed on duodenal mucosal proteins that were significantly correlated with the abundances of cSI-I (i) or cSI-N (ii) bacterial taxa (*clusterProfiler*).

Supplement 9

Supplement 10

List of Supplementary Materials

Materials and Methods

Figs. S1 to S10

Supplementary Results

Tables S1 to S10

**Table S5. Analyses associating fecal bacterial taxa with plasma proteins in women. (A,B)** Plasma proteins (A) and fecal bacterial taxa (B) predictive of cohort membership (DIABLO). **(C)** Results of linear models relating relative abundances of bacterial taxa with plasma protein levels. **(D,E)** Enrichment analysis performed on plasma proteins positively (D) or negatively (E) associated with bacterial abundance (*gProfiler2* over-representation test). **(F)** Enteropathogens detected in fecal samples using a microfluidics-based qPCR platform.

**Table S6. Bacteria cultured from duodenal aspirates of women in the Low BMI and Well-nourished cohorts. (A)** Summary of the number of isolates obtained per species. **(B)** Isolates with their 16S rRNA sequences (Sanger) and taxonomic identification (GreenGenes; 2024 release).

## Figures and Tables

**Fig. 1.**
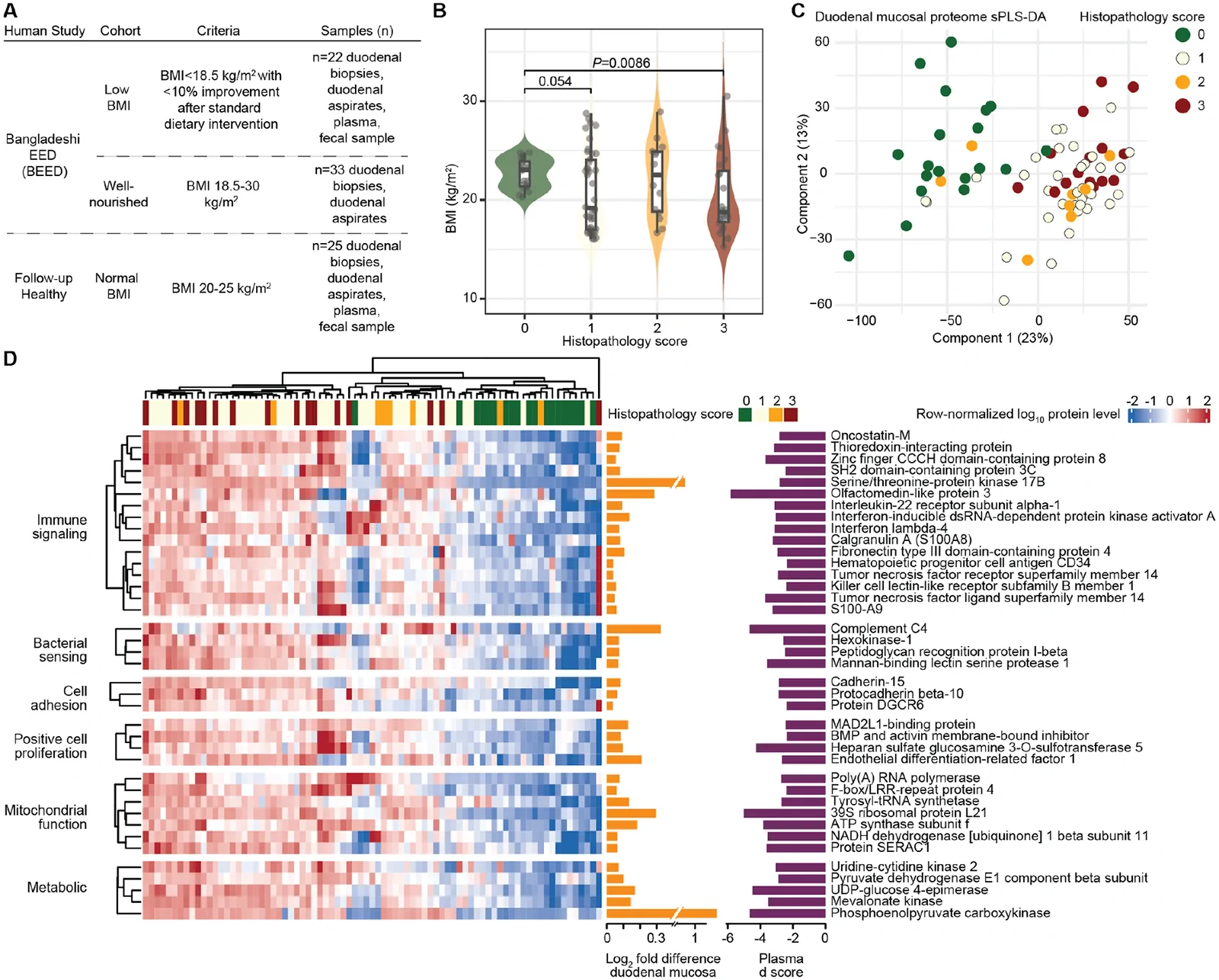
Undernutrition in adult Bangladeshi women with histopathologic evidence of EED is characterized by intestinal and systemic inflammation. **(A)** Human studies investigating the microbial contribution to undernutrition in adult women; overview of human biospecimens collected. **(B)** Distribution of BMI for participants within each histopathology score (Kruskal Wallis *P*=0.067, *P*-values shown are the result of Wilcoxon rank-sum tests). **(C)** Dimensionality reduction of sparse partial least-squares discriminant analysis (sPLS-DA) of duodenal mucosal proteomes (n=79 women). Color denotes histopathology score. **(D)** A subset of the 84 proteins whose levels were significantly higher in undernourished women with EED compared to healthy controls in both plasma (SAM *q*<0.05, n=47 women) and duodenal mucosal biopsies (*limma* adjusted *P*-value<0.05, n=79 women). The heatmap depicts log_10_-transformed, row-normalized levels in the duodenal mucosa. Bar plots show the magnitude of differences in biopsies and plasma. See table S1D for a full list of protein annotations and their thematic groupings.

**Fig. 2.**
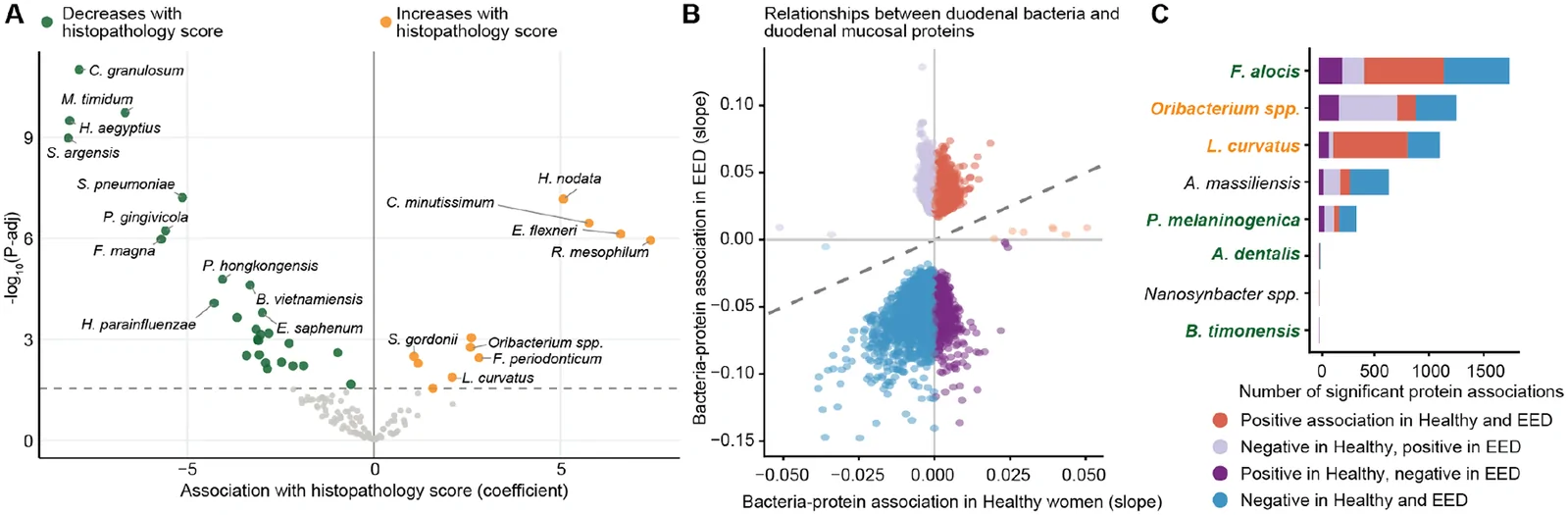
Duodenal microbiota and its associations with the duodenal mucosal proteome in women with EED. **(A)** Bacteria whose absolute abundances in duodenal aspirates increased (positive, orange) or decreased (negative, green) with histopathology score (zero-inflated Gaussian mixture model). **(B)** ß coefficients for linear models relating duodenal aspirate bacterial abundance to duodenal mucosal protein levels. Bacteria-protein linear relationships that differed significantly between women with and without EED were included and are colored according to the direction of their association (*aptamer ~ bacterial_abundance + EED_status + bacterial_abundance:EED_status*). **(C)** Bacteria that exhibited the greatest number of significant associations with duodenal mucosal proteins in women with EED compared to healthy controls (linear models described in (B)). As in (A), orange indicates taxa whose abundances increased significantly with histopathology score; green, taxa whose abundances decreased with histopathology score or were significantly elevated in women lacking histopathologic evidence of EED (**table SC**). For panels (A), n=80 women, for panels (B-C), n=79 women.

**Fig. 3.**
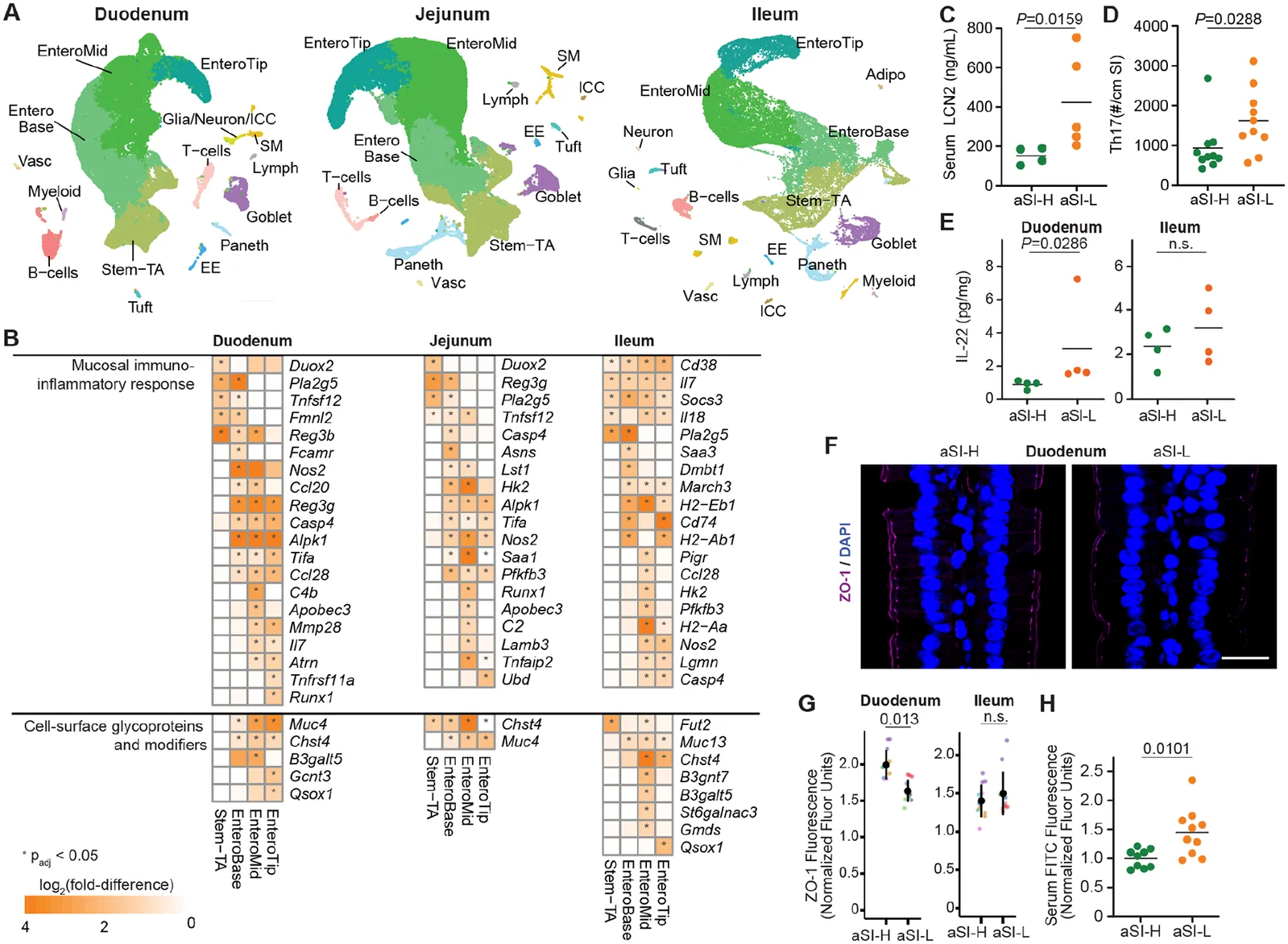
Duodenal bacteria cultured from Low BMI women with EED impair gut barrier function and induce inflammatory epithelial signaling in adult female mice. **(A)** Uniform Manifold Approximation and Projection (UMAP) dimensionality reduction of snRNA-Sequencing in duodenal, jejunal, and ileal tissue from aSI-L and aSI-H adult female mice (n=4 mice/intestinal segment/group). Adipo, adipocyte; EE, enteroendocrine; EnteroBase, EnteroMid, EnteroTip, enterocytes from villus base, mid-villus, and villus tip; ICC, interstitial cells of Cajal; Lymph, lymphatic endothelial cells; SM, smooth muscle; Stem-TA, intestinal stem cell and transit amplifying cells; Vasc, vascular endothelial cells. **(B)** Genes involved in mucosal immuno-inflammatory responses as well as genes encoding cell surface glycoproteins and their modifiers whose expression was significantly higher in intestinal epithelial nuclei of aSI-L compared to aSI-H adult female mice (*DESeq2* Wald test with Benjamini-Hochberg correction, ‘pseudo-bulk’ differential expression). **(C)** Serum LCN2 levels (n=4–5 mice/group, bars denote means). **(D)** Proportions of T cells in the small intestinal lamina propria (n=10 mice/group, bars denote means). **(E)** Levels of IL-22 in homogenized duodenal and ileal tissue (n=4 mice/group, bars denote means). **(F)** Representative immunofluorescent images for staining of zonula occludens-1 (ZO-1) in the duodenum of adult female mice colonized with the aSI-L or aSI-H consortia (scale bars=8 μm). **(G)** Mean fluorescence intensity (n=3 villi/segment/mouse for n=4 mice/group, mean ± s.d. shown). A linear mixed-effects model *(Measure ~ Microbiota + (1 | Mouse ID)*) was used to assess statistical significance; each color denotes a different mouse. **(H)** FITC-4 kDa dextran in serum 4 hours after gastric gavage (n=9–10 mice/group, bars denote means). For **C, D, E** and **H,** Wilcoxon rank-sum tests.

**Fig. 4.**
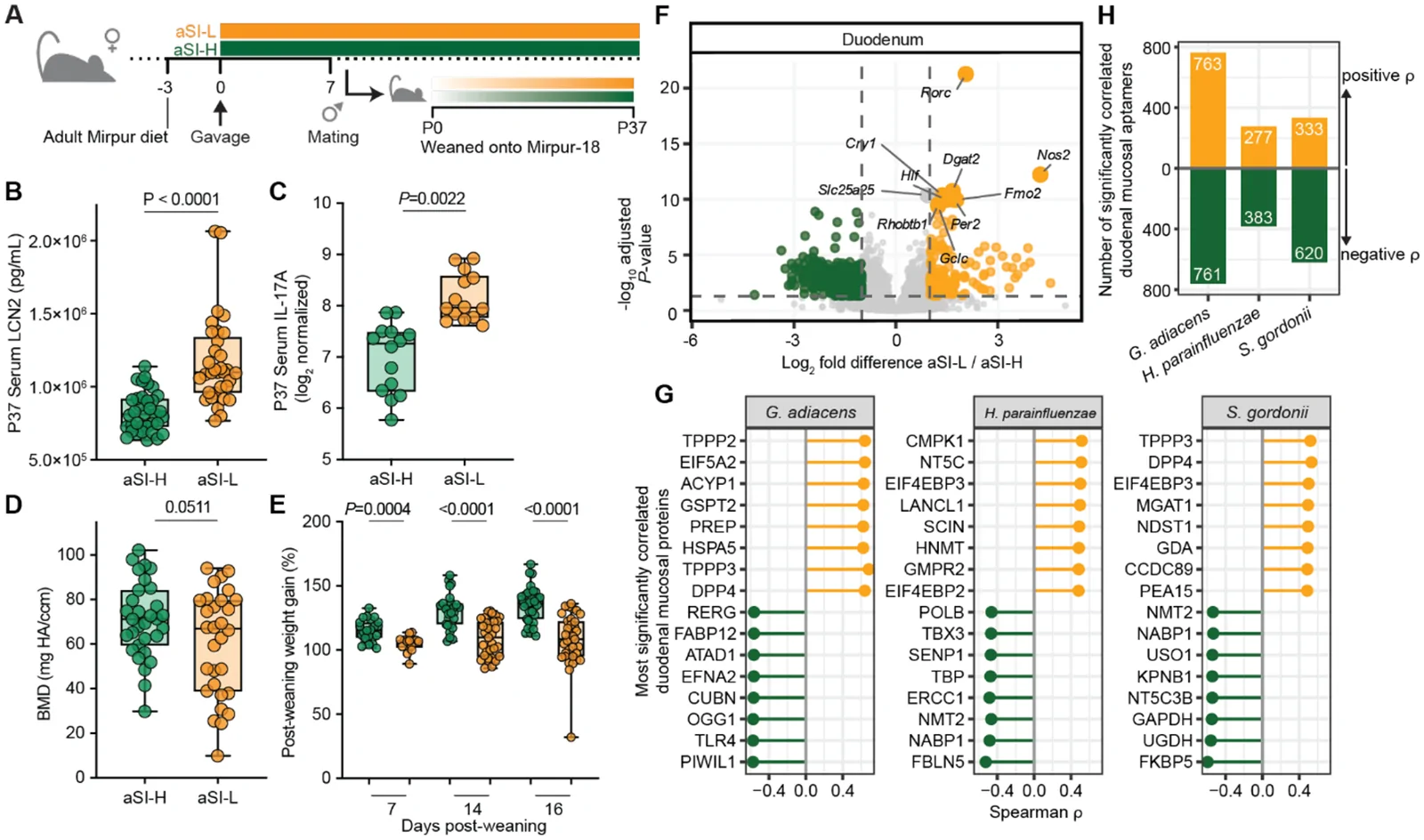
Effects of intergenerational transmission of aSI-L and aSI-H consortia in P37 offspring. **(A)** Experimental design; numbers denote experimental day relative to timing of gavage. **(B)** Serum LCN2 levels in P37 offspring (n=33–35 animals/group). **(C)** Serum IL-17A levels in P37 offspring (n=14 mice/group). **(D)** Femur bone mineral density in P37 offspring (n=30–31 animals/group). **(E)** Pup post-weaning weight gain (n=35 animals/group; pups were weaned at P21). For panels (B-E), each dot represents an individual animal. **(F)** Significantly differentially expressed genes in the duodenum of P37 aSI-L (positive, orange) compared to P37 aSI-H (green, negative) offspring (*DESeq2* Wald test, n=16 animals/group). **(G-H)** aSI-L bacteria transmitted to P37 offspring that were identified in the duodenal aspirates of women and correlated with proteins in their duodenal biopsies. **(G)** The 8 duodenal mucosal proteins in women that were most significantly positively and negatively correlated with the absolute abundances of *G. adiacens, H. parainfluenzae* and *S. gordonii* in aspirates (Spearman correlations, Benjamini Hochberg-adjusted *P*-values, n=79 women). **(H)** The total number of significant positive and negative correlations between the absolute abundances of *G. adiacens, H. parainfluenzae* and *S. gordonii* in duodenal aspirates and protein levels in the duodenal mucosa (Spearman, Benjamini Hochberg-adjusted *P*-values, n=79 women).

**Fig. 5.**
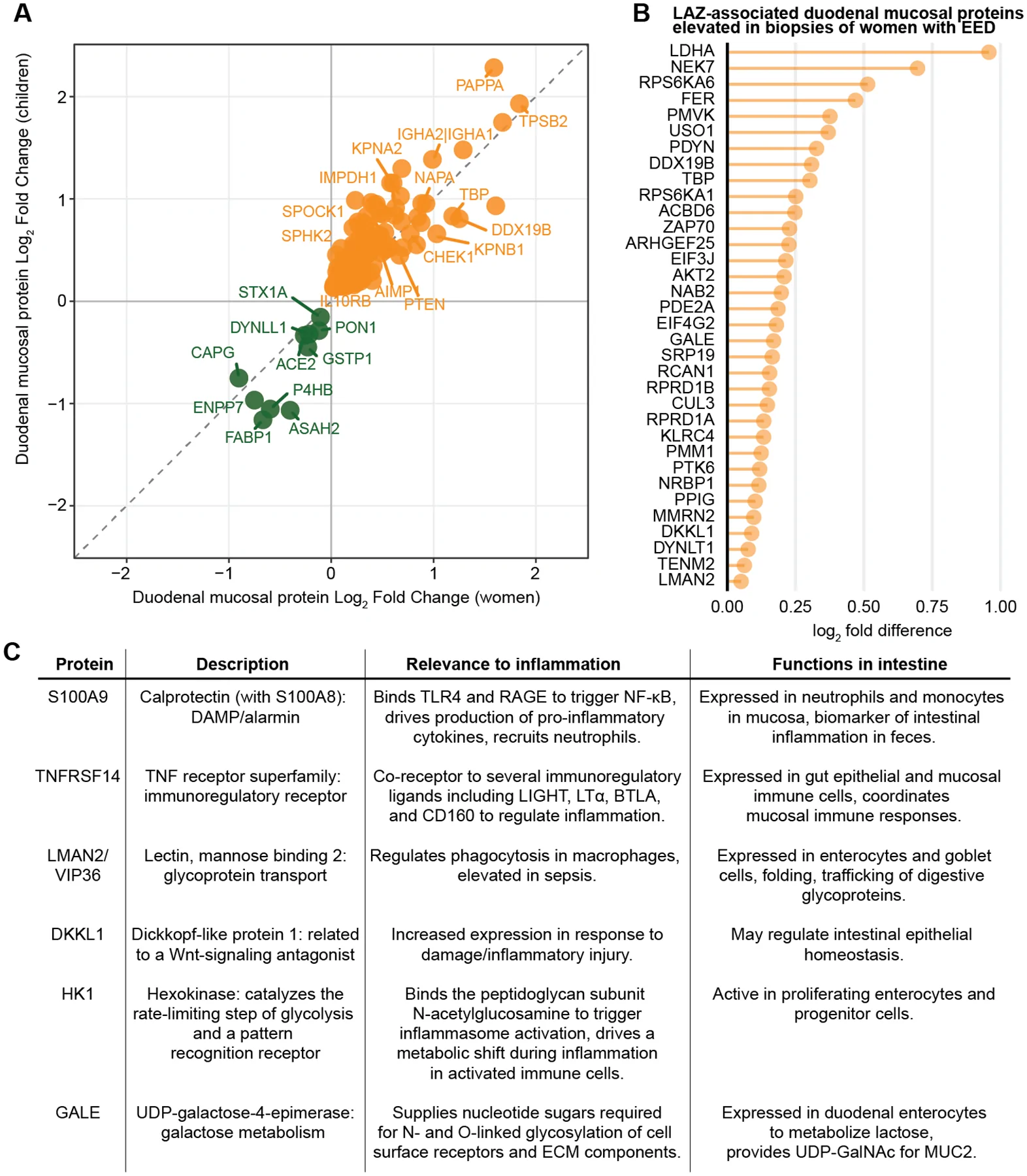
Duodenal mucosal proteins indicative of EED in women and children. **(A)** Concordance between duodenal mucosal proteins whose levels were significantly higher (positive, orange) or lower (negative, green) in women and children with severe EED (histopathology score 3; n=16/79 biopsies from women in the current study, 37/89 undernourished children in the BEED study (14) whose LAZ failed to improve after nutritional intervention) compared to those without histopathologic evidence of EED (histopathology score 0; n=20/79 women in the current study, 5 out of the 89 children (14), *limma eBayes*). **(B)** Duodenal mucosal proteins that were negatively correlated with length-for-age z-score (LAZ) in children with EED (14) and whose levels were significantly higher in biopsies of women with EED (histopathology score ≥ 1, n=59/79 women) versus those without EED (histopathology score 0, n=20/79 women). **(C)** Functional annotations of proteins whose levels were elevated in the duodenal mucosa of undernourished Bangladeshi women and children with EED (as in Fig. 5AB) as well as in the plasma of undernourished women (table S1D).

## Data Availability

DNA sequencing, bulk RNA-seq, and snRNA-seq data have been deposited in NCBI’s Sequence Read Archive (SRA) under project number PRJNA1489488 and will be made publicly available prior to publication. This study did not use any original code. Additional information required to reanalyze the data reported in this paper is available from the lead contact (jgordon@wustl.edu) upon request.
