# Supplementary material for: Functional characterization of duodenal microbiota and associated enteropathy in undernourished Bangladeshi women and gnotobiotic mice
Source: medRxiv. 2026 Sep 4:2026.08.14.26360472. Preprint. [Version 2] doi: 10.64898/2026.08.14.26360472 (PMC13684689; doi:10.64898/2026.08.14.26360472)
Supplement: Supplementary file 1 [file media-11.pdf]

Supplementary Materials and Methods

HUMAN STUDIES

Sequencing full-length 16S rRNA amplicons from human fecal samples

DNA from frozen fecal samples was extracted as described previously *(61)*. Briefly, fecal samples were pulverized in liquid nitrogen, ~50 mg of pulverized material was homogenized by bead-beating with 500 µL 0.1 mm zirconia silica beads in a solution of 500 µL phenol:chloroform:IAA (25:24:1), 210 µL 20% SDS and 500 µL buffer A followed by purification (QiaQuick, Qiagen) and storage in Tris-EDTA buffer.

Fecal DNA samples were normalized to a concentration of 0.4 ng/µL. Libraries were prepared and analyzed as described in '*Sequencing full-length 16S rRNA amplicons generated from duodenal samples*' with the following modifications: reads were not normalized to exogenous spike-in counts. Differential abundance testing at the species level was performed using Benjamini Hochberg-corrected Wilcoxon rank-sum tests and Kruskal Wallis followed by Tukey's post-hoc tests.

Enteropathogen detection by multiplex quantitative PCR

DNA extracted from fecal samples obtained from undernourished women or healthy controls was quantified via Qubit then normalized to 2 ng/µL. Levels of 19 common bacterial, viral, and protozoal gastrointestinal pathogens were determined with a microfluidic digital PCR system (Fluidigm Juno). Previously described primers *(21)* that target unique sequences within each pathogen were used; mean C_t values < 30 designated a positive result (i.e., presence). Pathogen prevalence is reported as presence/absence in **Table S5F**.

Dimensionality reduction of human bacterial and proteomic datasets

Dimensionality reduction of duodenal and fecal bacterial taxa was applied using the R package *mixOmics* (v6.28.0) *(62)*. Sparse partial least-squares discriminant (sPLS-DA) *(20)* was used to predict taxa (ASVs) that were associated with cohort (fecal samples) or histopathology score (duodenal aspirates). Bacterial abundances were centered and log ratio-transformed using a tuning step with M-fold validation as follows: *tune.splsda(ncomp=6, logratio='CLR', validation='Mfold', folds=5, nrepeat=50).* The number of features (bacterial species) to test from each dataset was defined with *c(1:10, seq(20, nASVs, 10))* where *nASVs* was the total number of species included in each analysis. The final sPLS-DA model was run with *splsda(logratio='CLR', ncomp, keepX)* where the number of components *ncomp* and number of features *keepX* were defined using the output of *tune.splsda*. The sPLS-DA models were evaluated with *perf(folds=5, validation='Mfold', dist='max.dist', nrepeat=50*).

A sPLS-DA model for duodenal mucosal proteins was fit using log_{10}-transformed data that were normalized as described in '*Duodenal and plasma proteomic analyses*', using biopsy histopathology score as the predictive variable *splsda(Histopathology_Score, ncomp=3)*.

Data Integration Analysis for Biomarker discovery using Latent cOmponents ('block' sparse partial least squares discriminant analysis)

Block sPLS-DA was used *(63)* to integrate bacterial abundance with proteomic data (bacterial abundance in duodenal aspirates with levels of duodenal mucosal proteins in matched

samples; bacterial abundance in fecal samples with levels of plasma proteins in matched samples). SomaScan datasets were filtered to remove the 30% of aptamers with the lowest variance then centered (*scale(center=T)*). Bacterial abundances were log ratio-transformed and centered; the 10% of species with lowest variance were removed. Clinical cohort (undernourished vs. healthy) was used as the categorical variable for the fecal bacteria-plasma proteome comparison while histopathology score was used for the duodenal aspirate bacteria-duodenal mucosal proteome comparison. The final block sPLS-DA models were run with *block.plsda(ncomp=5, design, ncomp, keepX)*, where *design* was created using the correlation between the X and Y features resulting from *pls(ncomp=1)* on the bacterial abundance and SomaScan matrices. The models were evaluated with *perf(validation='Mfold', folds=10, nrepeat=10)*, which was used to provide the optimal number of components, *ncomp.* The number of features (*test.keepX*) to include in the test was defined with *c(seq(10,250,25))* for bacteria and *c(seq(10,1000,100))* for aptamers. Block sPLS-DA models were tuned with *tune.block.splsda(ncomp, test.keepX, design, validation='Mfold', folds=f, nrepeat=1, dist='centroids.dist')*, where *f=8* for the duodenal bacteria-proteome model and *f=10* for the fecal bacteria-plasma proteome model. The tuning function provided the number of bacterial species and aptamers (features in *keepX*) to retain in the final model.

<u>Culturing bacteria from duodenal aspirates</u>

The methods used for culturing bacterial strains from duodenal aspirates obtained from adult women enrolled in the BEED study were analogous to those we employed previously *(14)*. All culturing materials were pre-reduced in a Coy chamber, under an atmosphere of 75% N_2, 20% CO_2 5% H_2, for at least 24 hours. Duodenal aspirates were thawed under anaerobic conditions on wet ice. A 100 µL aspirate sample was diluted 1:4 in 300 µL 1X PBS. 100 µL aliquots of diluted aspirate were plated on two Brain Heart Infusion plates supplemented with 10% defibrinated horse's blood (BHI-BA) and two Chocolate Agar plates (Hardy Diagnostics, H25). One BHI-BA plate and one Chocolate Agar plate were incubated at 37 ^{o}C under anaerobic conditions. The other two plates were grown at 37 ^{o}C under microaerophilic conditions (NuAire In-VitroCell Direct Heat CO_2 incubator, 85% N_2, 10% CO_2, 5% O_2). Every 24 hours, colonies were picked into 600 µL of BHI broth in a 96-well plate containing 1 mL wells. After 48 hours growth at 37 ^{o}C under the same atmospheric conditions, strains were stored at -80 ^{o}C in 100 µL of a 1:1 mixture of 30% glycerol (prepared with 1X PBS).

***Pooling* –** Strains were stored in 96 well plates at -80 ^{o}C in PBS containing 15% glycerol (v/v). For isolates originally isolated under anaerobic conditions, plates were thawed under anaerobic conditions and 20 µL aliquot of each stock was used to inoculate a well in a 1 mL deep-well plate (Thermo Scientific) containing 600 µL BHI broth (followed by anaerobic growth at 37 ^{o}C. For isolates originally isolated under microaerophilic conditions, a 20 µL aliquot of each stock was inoculated into 600 µL BHI broth prior to microaerophilic growth at 37 ^{o}C.

After incubation for 72 hours at 37 ^{o}C, a 20 µL aliquot of each anaerobic or microaerophilic culture was added to 600 µL fresh medium (sub-culture) which was incubated for an additional 48 hours at the same temperature under the same atmospheric conditions. Equal volumes of each isolate sub-culture were then pooled in 50 mL conical tubes anaerobically and the mixture was centrifuged at 2,500 x *g* for 15 minutes. Under anaerobic conditions (75% N_2, 20% CO_2 5% H_2), the supernatant was removed, the pellet was resuspended in 30% glycerol, using 1/5 of the original culture volume and the pooled culture collections were aliquoted into 1.8-mL crimp glass vials (Wheaton), which were sealed and stored at -80 ^{o}C.

Isolate identification – Individual bacterial isolates were identified by Sanger sequencing of full-length 16S rRNA amplicons generated by primers 8F and 1391R. An aliquot of each isolate's broth culture that had been stored in PCR plates at -20 °C were thawed; 5 µL of that aliquot was transferred to a separate PCR plate and 45 µL 'lysis buffer' (25 mM NaOH, 0.2 mM EDTA) was added. The PCR plate was sealed with aluminum tape, incubated at 95 °C for 30 minutes then centrifuged briefly at 1,000 x g to gather condensate. 50 µL of 'neutralization buffer' (0.040M Tris-HCl) was added to the culture with lysis buffer. The plate was then centrifuged for 6 minutes at 3,000 x g. For each sample, 12.5 µL of One Taq Hot Start 2X (New England Biolabs M0484S), 0.5 µL of both 8F and 1391R primers (10 µM), and 9.5 µL of nuclease free water were added to 2 µL of lysed template DNA. PCR was performed using a Bio Rad C1000 Touch Thermal Cycler and the following cycling conditions: 30 seconds at 94 °C for the initial denaturation, followed by 34 cycles of 15 seconds at 94 °C (denaturation), 15 seconds at 52 °C (annealing), and 90 seconds at 68 °C (extension). This was followed by a 5-minute final extension at 68 °C.

Successful generation of PCR amplicons was confirmed by gel electrophoresis using a 1.5% agarose gel in 1 x TAE buffer with 1 kb DNA ladder for reference. Samples with visible bands were sent to Azenta Life Science for enzymatic PCR clean up and Sanger sequencing. Raw sequences were trimmed and aligned with Geneious Prime (http://www.geneious.com). Trimmed reads were then assembled *de novo* using Geneious Prime's 'De Novo Assemble' function. Taxonomic assignments were based on alignments obtained with BLAST utilizing the NCBI database. The best quality hit was selected based on the 'Grade' score given by the software; this score considers query coverage, e-value, and identity values. Only scores with a 'Grade' above 90% were considered. The isolate with the best quality hit for each unique species type was then selected for generation of permanent stocks.

GNOTOBIOTIC MOUSE EXPERIMENTS

Husbandry for initial tests of duodenal bacterial culture collections

4-5-week-old germ-free C57Bl/6J mice were given *ad libitum* access to a standard chow diet (Diet 2018S, Envigo). Three days prior to colonization, mice were switched to the Adult Mirpur diet and were given *ad libitum* access throughout the course of the experiment. For initial characterizations of gnotobiotic mice orally gavaged (oral gavage needle; Cadence Science; catalog no. 7901) with the pooled Low BMI or Healthy BMI culture collections, animals were weighed and fecal samples were collected in the gnotobiotic isolators. Animals were euthanized 18 days after colonization and LCN2 and MMP8 levels were assessed in serum (see *Protein measurements from serum and intestinal tissue*).

Generation of aSI-L and aSI-H consortia

Flash-frozen cecal contents stored at -80 °C from mice with the highest and lowest levels of serum LCN2 that were colonized with the pooled Low BMI and Healthy BMI culture collections were selected for clarification. Individual mouse cecal contents were weighed and homogenized in pre-reduced 30% glycerol prepared in 1X PBS and supplemented with 0.05% L-cysteine; homogenization was achieved with 2 mm glass beads under anoxic conditions in a 50 mL conical tube. After filtration through a 100 µM cell strainer (Corning) 30% glycerol was added to a final concentration of 1% w/v. These clarified cecal contents from individual mice were aliquoted to 2 mL crimp top glass vials under anaerobic conditions prior to storage at -80 °C.

Husbandry for intergenerational transmission experiment

6-8-week-old germ-free C57Bl/6J mice were given *ad libitum* access to an autoclaved breeder chow (Lab Diet 5021; Purina Mills, Richmond, IN) until 3 days prior to colonization, at which time they were switched to the Adult Mirpur diet for the remainder of the experiment. Mice received 200 µL of the stock solutions of the aSI-L or aSI-H consortia via an oral gavage needle.

One week after initial gavage, trio matings were performed (two females with one male). Pups born to these mothers were maintained with their dams in the same cage until weaning on postnatal day 21 (P21), at which time pups from the same litter were transferred to new cages and weaned onto the Mirpur-18 diet. For dams and their pups, bedding was replaced every 7 days and diets were provided *ad libitum*. Following weaning at P21, offspring weights were monitored and fecal samples were acquired.

Tissue collection from P37 offspring – At the time of euthanasia, blood was collected retro-orbitally in serum separator microtainer tubes (BD) and kept at room temperature prior to centrifugation at 2,000 x *g* for 10 minutes; separated serum was aliquoted into 2 mL screw cap tubes and stored at -80 °C.

The intestine was removed and dissected on a disposable dissection board placed on wet ice. The small intestine was divided into thirds (approximating the duodenum, jejunum, and ileum) and intestinal contents from each portion of the small intestine and colon were removed by gentle extrusion with curved forceps, weighed in 2 mL screw cap tubes, and snap-frozen in liquid nitrogen. Cecal contents were sampled using 10 µl plastic loops, frozen in liquid nitrogen then placed in 2 mL screw cap tubes. From the most proximal end of each portion of the small intestine, three 1 cm tissue segments were acquired, which, in addition to the remaining tissue segment, were stored in separate 2 mL screw cap tubes, flash-frozen in liquid nitrogen prior to storage at -80 °C. For the colon, two 1.5 cm segments were collected plus the remaining colonic tissue.

Tissue collection from adult female mice – Serum was collected as described above. The intestine was removed and dissected on a disposable dissection board placed on wet ice. The following sections of the duodenum, jejunum, and ileum were flushed with PBS and pinned out in 10% neutral buffered formalin for histologic assessment: 3-5 cm from the gastroesophageal junction (GEJ), 15-17 cm from the GEJ, and 3-5 cm from the ileal-cecal valve (ICV). The following sections of the duodenum, jejunum, and ileum were cleared of intestinal contents with gentle extrusion with curved forceps then flash-frozen in liquid nitrogen and stored at -80 °C for snRNA-seq: 2-3 cm from the GEJ, 14-15 cm from the GEJ, and 1.5-3 cm from the ICV. Sections of the duodenum (1-2 cm from the GEJ) and ileum (6-7.5 cm from the ICV) were cleared of intestinal contents and flash frozen for protein assays.

FITC-4 kDa dextran gut permeability assay

After 4 hours of fasting, mice were gavaged with 80 mg/mL FITC-4 kDa dextran (Sigma Aldrich) at 0.6 mg/g. Food and water were withheld for an additional 4 hours, followed by collection of blood and euthanasia. Serum was isolated with a BD microtainer and fluorescence was measured using an excitation wavelength of 485 nm and emission wavelength of 530 nm with the Tecan Spark 20M plate reader.

Micro-computed tomography (µCT) of bone

The femur was harvested from the left rear leg of P37 offspring and cleaned of muscle and connective tissue. Femurs were stored in 10 mL 10% neutral buffered formalin at room temperature for 24 hours. Fixed femurs were then washed in 1X PBS for 15 minutes; this wash step was repeated two more times, followed by a 30% ethanol wash for 30 minutes, a 50% ethanol wash for 30 minutes and a final 70% ethanol wash for 30 minutes. Femurs were subsequently embedded in 2% agarose and scanned with a µCT 40 desktop cone beam instrument (ScanCO Medical, Brüttisellen, Switzerland). For analyses of cortical bone, 100 slices were taken for each sample in the transverse plane, with a 6 µm voxel size (high resolution); slices began at the midpoint of the femur and extended toward the distal femur. The boundaries and thresholds for bone were drawn manually using µCT 40 software. Volumetric parameters were quantified using software associated with the ScanCO instrument.

Histomorphometric and immunohistochemical analysis of intestinal tissue

Segments from the duodenum, jejunum, and ileum (see 'Tissue collection from adult female mice') were fixed in 10% neutral buffered formalin for 1 day, then washed and stored in 70% ethanol, all at 4°C. Tissue segments were positioned in agar then embedded in paraffin followed by preparation of 5 µm sections on slides that were stained with hematoxylin and eosin then scanned with a Hamamatsu NanoZoomer at 20X resolution for histomorphometric measurements of 10 well-oriented villus-crypt units/tissue segment/mouse using *QuPath (64)*. For immunostaining, additional 5 µm sections on slides were prepared and deparaffinized with successive immersion in xylene, ethanol, and water mixtures (*i.e.* 100% xylene, 50% xylene/50% ethanol, 100% ethanol, 95% ethanol, 70% ethanol, 50% ethanol, and water). Slides were then boiled in Tris-EDTA Buffer pH 9.0 buffer (Abcam, ab93684) for antigen retrieval, blocked with 10% normal goat serum (Abcam, ab7481) for 2 hours at room temperature, and incubated overnight at 4 °C with anti-ZO-1 primary antibody (1:100, ThermoFisher Scientific #61-7300) diluted in antibody diluent (Abcam, ab64211). Sections were subsequently washed with TBS-Triton-X 100 and incubated with goat anti-rabbit IgG–Alexa Fluor 647 (1:500, ThermoFisher Scientific #A-21245) for 2 hours. Stained sections were mounted with Fluoroshield Mounting Media containing DAPI (Abcam, ab104139) and scanned with a 3DHistech P250. Sections were analyzed in a blinded fashion using *QuPath (64)* where villus peripheries were traced (3 villi/intestinal segment for n=4 mice/treatment group) and exported to *FIJI (65)* for quantification of mean fluorescence intensities and normalization to background fluorescence intensities. Representative high-resolution images were collected with the Zeiss LSM 880 microscope (under 63X magnification/1.4 NA oil).

Protein measurements in serum and intestinal tissue

Serum (flash-frozen upon isolation with a BD microtainer after mouse sacrifice) was thawed on ice and diluted 1:1000 for use with the Mouse Lipocalin-2/NGAL DuoSet ELISA kit (R&D Systems), 1:30,000 for use with the Mouse Complement C3 ELISA kit (Abcam), or subjected to the Mouse Cytokine/Chemokine 32-Plex Discovery Assay® Array (MD32) for measurement of IL-6 and IL-17A (Eve Technologies) or an O-link Target 96 Mouse Exploratory assay (Thermo Fisher; see **Table S9A** for list of proteins assayed). Frozen intestinal tissue was homogenized by (i) adding a 20-30 mg segment from the duodenum or ileum (see 'Tissue collection from adult female mice'), to a vial with 1.6 mm aluminum oxide and silicon carbide particles (Matrix F; MP Biomedicals) and 600 µL cold T-PER buffer (Thermo Fisher Scientific) with Complete Ultra protease inhibitor (Roche) followed by (ii) bead beating for 1 minute then centrifugation at 13,000 x g for 5 minutes at 4 °C. Protein concentrations in the resulting

supernatants were measured (Pierce BCA Protein Assay Kit; Thermo Fisher Scientific) and adjusted to 1 mg/mL using sterile PBS with protease inhibitor (Roche). IL-22 levels were measured with the Mouse Cytokine Th17 12-Plex Discovery Assay® Array (MDTH17-12, Eve Technologies).

Flow cytometry of immune cell populations

Mice received a retro-orbital injection of 2 µg of anti-CD45 antibody to label intravascular leukocytes. Animals were euthanized 3 minutes post injection and small intestinal leukocytes were isolated as previously described *(66)*. Briefly, small intestines were flushed to remove luminal contents, and Peyer's patches and mesenteric fat were removed. Tissues were opened longitudinally and incubated for 20 min with gentle agitation in Hanks' balanced salt solution (HBSS) supplemented with 1 M HEPES (Corning), 10% bovine calf serum (HyClone), and 0.5 M EDTA (Corning) to remove epithelial cells, followed by vortexing. After a second round of gentle agitation and vortexing in fresh HBSS/bovine calf serum/EDTA buffer, the tissue was washed with HBSS and digested in Complete RPMI 1640 medium (Gibco) containing collagenase IV (Sigma–Aldrich) for 40 minutes at 37 °C with agitation. The resulting cell suspension was filtered through a 100-µm strainer (Falcon) and leukocytes were enriched by density gradient centrifugation using 40% and 70% Percoll (GE Healthcare). Cell suspensions were incubated with Fc block (clone 2.4G2, made in-house from HB-I97 hybridoma cells), treated with a viability dye (Live/Dead Aqua, Thermo Fisher Scientific), then subjected to staining for surface markers. The Foxp3 Staining buffer set (eBioscience) was then applied, followed by staining of intracellular proteins. The antibodies used are listed in **Table S8G**. Samples were analyzed using the BD FACSymphony A3 flow cytometer; the gating strategies shown in ***Supplementary Results*** and *FlowJo* (Treestar).

Bulk RNA-Seq of duodenum, ileum and colon from P37 mice

RNA extraction and library preparation – RNA was extracted from 1-1.5 cm flash-frozen segments of the duodenum, ileum and/or colon (piece #1, *see "Tissue collection from P37 offspring" above*) using the Qiagen RNeasy 96 Kit. Total RNA was quantified (Qubit) and quality was assessed using a TapeStation (Agilent). cDNA libraries were generated using the Illumina Total RNA Prep with Ribo-Zero kit (Illumina). Barcoded libraries were sequenced on Illumina NovaSeq X Plus instrument [150 nt paired-end reads to a depth of $1.2x10^7 \pm 2.2x10^6$ reads/sample (mean ± s.d.).

Data analysis – Read quality was verified with *FastQC* (v0.11.7; http://www.bioinformatics.babraham.ac.uk/projects/fastqc/). Reads were trimmed to remove adapters and low-quality read segments using *trimgalore* (*trimgalore –quality 20 –fastqc*, v0.6.6; https://github.com/FelixKrueger/TrimGalore). Trimmed reads were pseudoaligned (*kallisto quant -b 100 –plaintext –bias –rf-stranded –pseudobam,* v0.48.0) to a *kallisto(67)* index built from the Gencode v37 *Mus musculus* reference genome (*kallisto index gencode.vM37.transcripts.fa.gz*). MultiQC *(68)* (v1.13) was performed on trimmed and mapped reads. Transcript counts were aggregated to gene counts. *Kallisto* 'estimated counts' values and transcripts per million (TPM) values were used to generate a gene-level expression matrix in R using *tximport (69)* (v1.32.0) and *biomaRt (70)* (v2.60.1). Gene annotations were acquired with *tximeta* [*tx2gene = tximeta(coldata, type = "kallisto', cleanDuplicateTxps = T, useHub = T)*]. The 'lengthscaledTPM' function in *tximport* was used to adjust estimated counts by gene length and abundance [*tximport(x, type = 'kallisto', tx2gene, countsFromAbundance =*

'lengthScaledTPM')]. Raw counts were imported [*tximport(x, type = 'kallisto', tx2gene, countsFromAbundance = "no")*] for *DESeq2 (71)* differential abundance analysis and filtered at a threshold of at least 10 counts across the minimum group size (n=16 animals). Differential expression on the resulting count matrix was performed for each tissue separately with *DESeq2* [*dds = DESeqDataSetFromMatrix(countData, colData*, *design = ~Microbiota),* where 'Microbiota' denoted aSI-L versus aSI-H animals. *dds = DESeq(dds), res = results(dds, contrast = c("Microbiota", "aSI_L", "aSI_H")*].

EntrezIds were mapped onto gene IDs using *AnnotationDbi* (1.66.0; https://github.com/Bioconductor/AnnotationDbi) and *org.Mm.eg.db* (v3.19.1) [*mapIds(org.Mm.eg.db, keys = row.names(countData), keytype = "SYMBOL", column = "ENTREZID")*]. GSEA was performed using *clusterProfiler(48)* (v4.12.6) querying the Gene Ontology (GO) [*gseGO(geneList, org.Mm.eg.db, ont = "ALL", minGSSize = 20, nPermSimple = 10000, pvalueCutoff = 0.1, by = 'fgsea', eps = 0)*] and Reactome pathway databases [*gsePathway(geneList, organism = "mouse", minGSSize = 10, nPermSimple = 10000, pvalueCutoff = 0.1, by = 'fgsea', eps = 0)*]. Human homologues to mouse genes that were differentially expressed in the duodenum, ileum and colon of P37 aSI-L and aSI-H offspring were identified using the R package *homologene* (https://github.com/cran/homologene) with the function *human2mouse(db = homologeneData).*

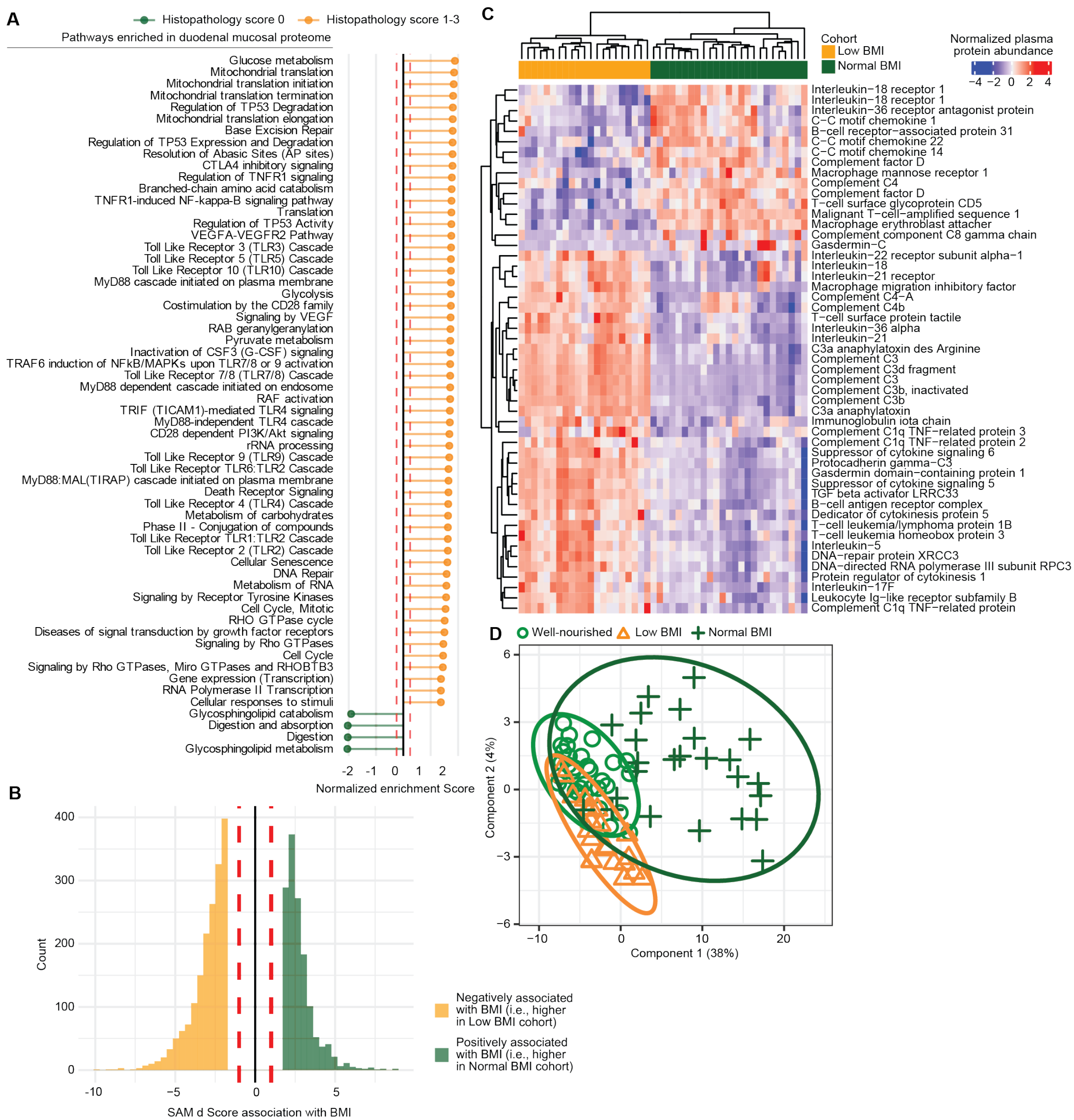

Fig. S1. Duodenal mucosal and plasma proteomic analysis of undernourished women with EED compared to healthy controls. (A) Reactome pathways enriched (GSEA) in the duodenal mucosal proteome of women with EED (histopathology score 1-3, n=59 out of 79 women) compared to healthy women (histopathology score 0, n=20 out of 79 women). **(B)** Plasma protein associations with BMI by significance analysis of microarrays (SAM, n=47 women). **(C)** Immuno-inflammatory proteins that were significantly associated with BMI. Values are row-normalized log_{10} values, top annotation denotes membership to clinical cohort (n=47 women). **(D)** Dimensionality reduction of bacterial composition within duodenal aspirates, colored by cohort (Partial least squares discriminant analysis, PLS-DA). Ellipses denote 95% confidence intervals (n=80 women total; n=22 Low BMI, n=33 Well-nourished, n=25 Normal BMI).

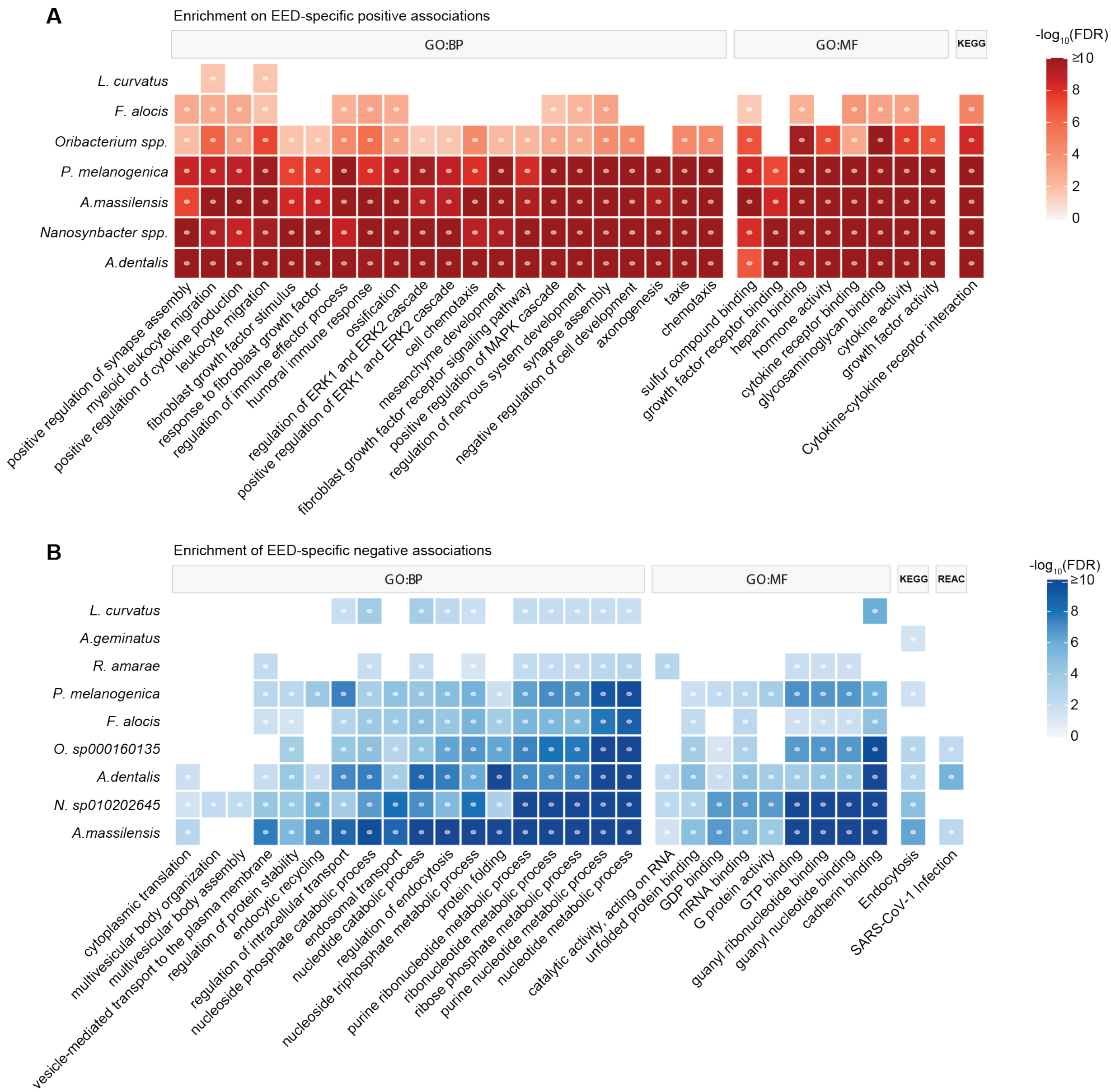

Fig. S2. Pathway enrichment analysis performed on duodenal mucosal proteins whose linear relationship with bacterial abundance differed significantly between women with and without EED. (A,B) Enrichment analysis was performed on proteins whose levels were significantly positively (A) or negatively (B) linearly related to the absolute abundances of bacteria in aspirates of women with EED, but not in healthy women. GO:BP, Gene Ontology Biological Process; GO:MF, Molecular Function; KEGG, Kyoto Encyclopedia of Genes and Genomes; ‘REAC’, Reactome database.

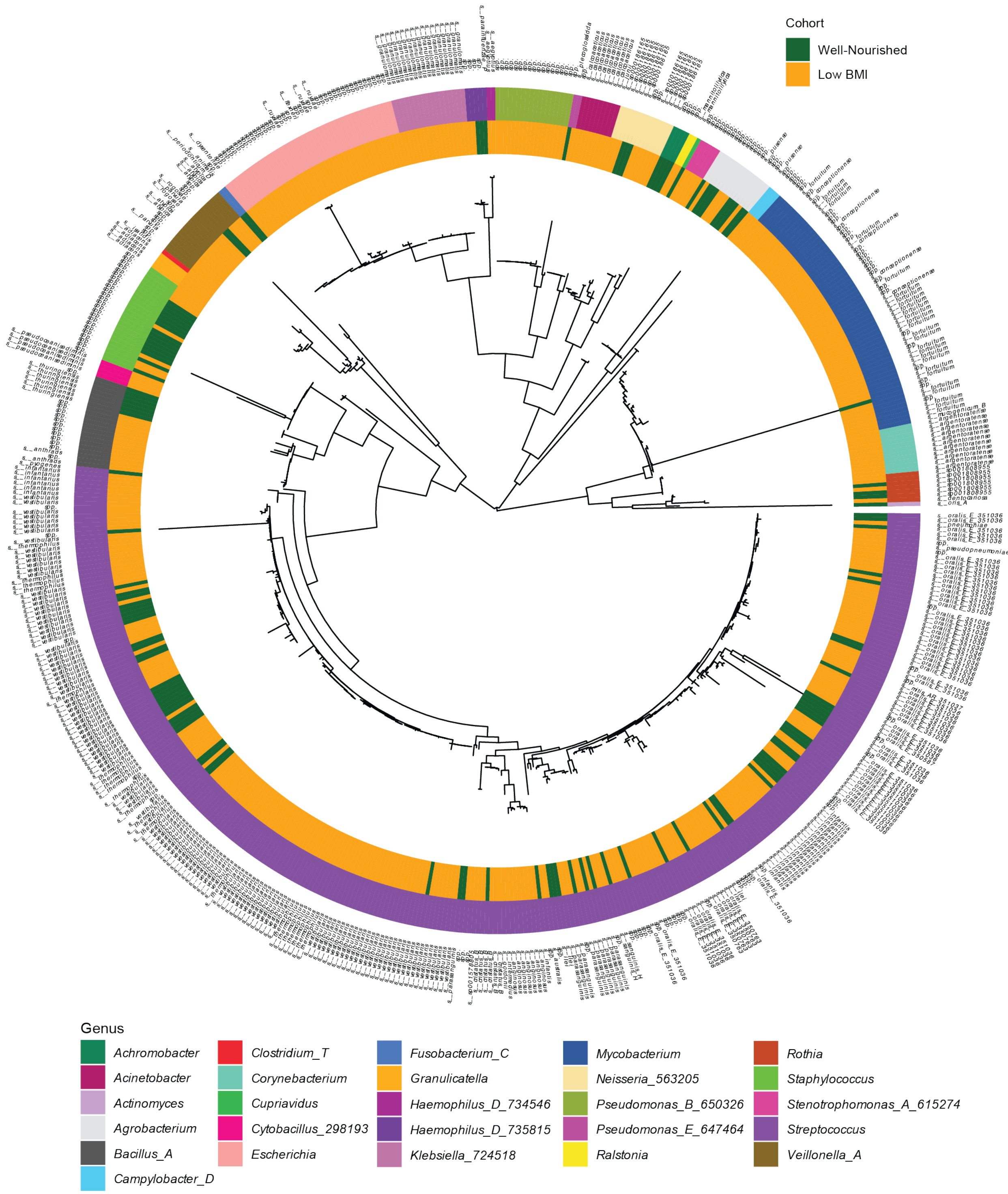

Fig. S3. Bacteria cultured from duodenal aspirates obtained from Bangladeshi women. Phylogenetic tree of the 480 isolates cultured from members of the Low BMI cohort (orange) and 125 isolates from women in the Well-nourished cohort (green). The outer colored ring indicates genus-level assignments; the text denotes species-level assignments.

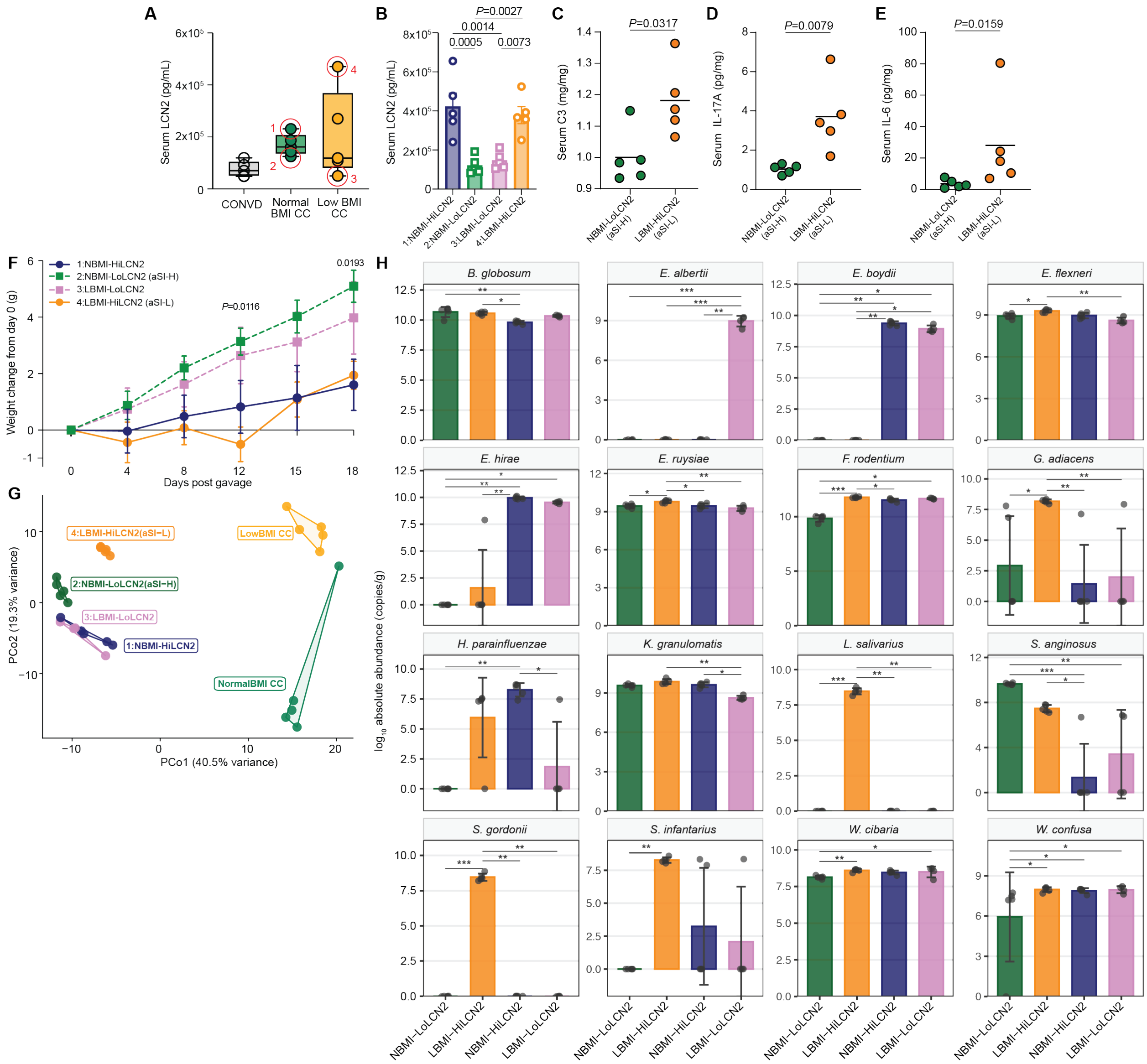

Fig. S4. Evaluating consortia of bacteria derived from undernourished and healthy women in gnotobiotic mice. (A) Serum LCN2 in mice colonized with intact culture collections pooled from Well-nourished donors (Healthy Culture Collection, 'H-CC'), Low BMI donors (Low BMI Culture Collection, 'L-CC'), or cecal contents acquired from a conventionally-raised mouse (CONV-D, boxes denote interquartile range). **(B)** Levels of serum LCN2 in groups of mice colonized with clarified cecal contents acquired from individual mice numbered 1-4 in panel a (mean ± s.d., one-way ANOVA with Tukey's post-hoc multiple comparisons). **(C-E),** Serum levels of complement protein C3 (C), IL-17A (D) and IL-6 (E) in groups of mice colonized with cecal contents acquired from animals 2 and 4 in panel (A) (bars denote mean, Wilcoxon rank-sum tests). **(F)** Post-colonization weight gain in groups of mice colonized with cecal contents that originated from animals 1-4 from (A) (repeated measures ANOVA, mean ± s.d.). **(G)** Principal coordinates analysis of bacterial composition in cecal contents of mice colonized with intact culture collections (L-CC, H-CC) or clarified cecal contents that originated from animals 1-4 in panel (A). **(H)** Absolute abundances of bacteria in the groups of mice gavaged with clarified cecal contents that originated from animals 1-4 in panel (A), Benjamini-Hochberg

corrected Wilcoxon rank-sum tests). For all panels, n=5 mice/group. Abbreviations used for panels (B-H): mice colonized with cecal contents obtained from mice harboring intact culture collections from Well-nourished (NBMI) or Low BMI (LBMI) donors that produced high (Hi) or low (Lo) levels of LCN2. NBMI-LoLCN2 is equivalent to ‘aSI-H’; LBMI-HiLCN2 is ‘aSI-L’.

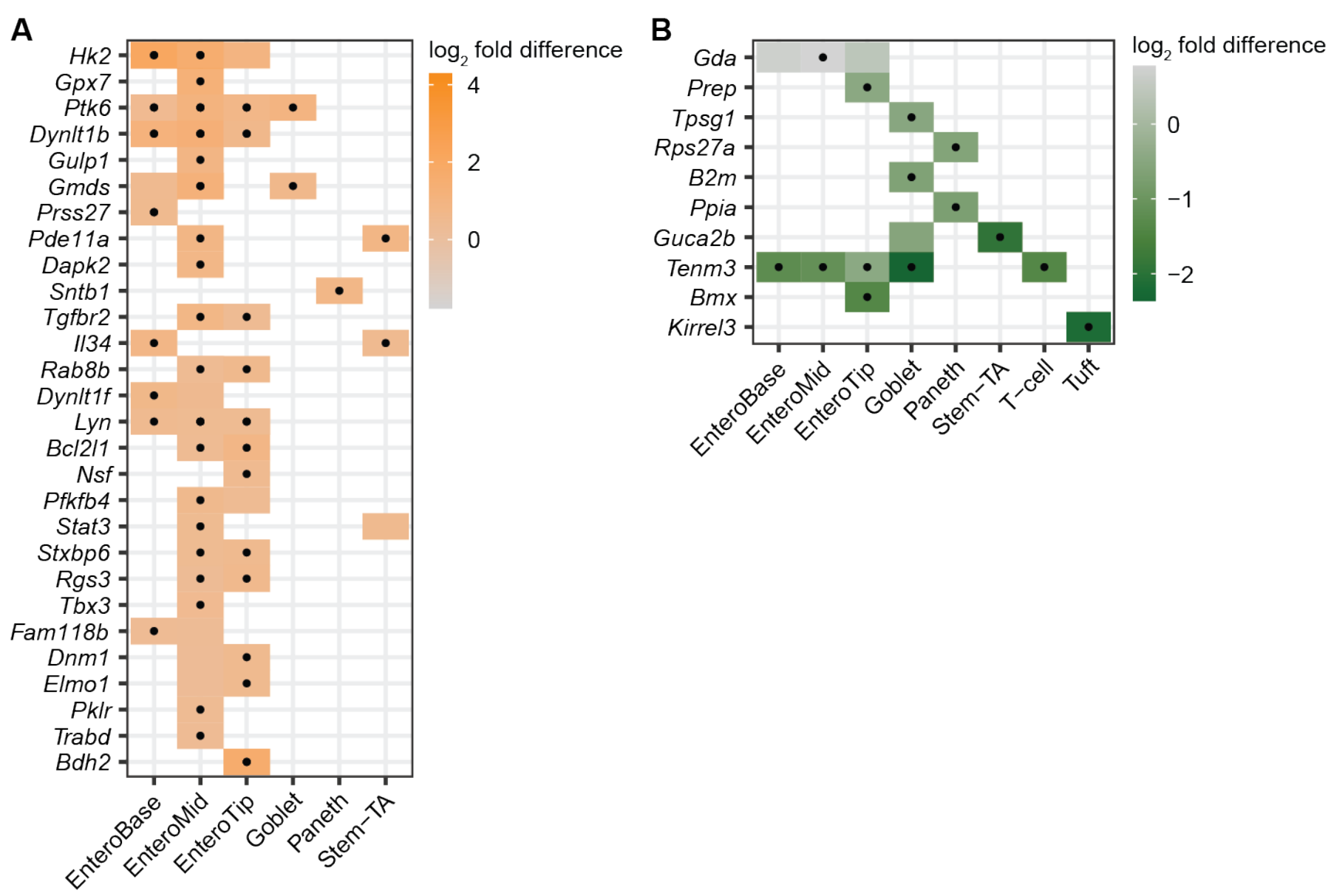

Fig. S5. Expression in the duodenum of adult female mice of mouse genes that are homologous to human duodenal mucosal proteins with significantly different levels between women with and without EED. Results from snRNA-Seq analysis of duodenal segments harvested from aSI-L and aSI-H adult females. **(A)** Genes that were significantly more highly expressed (*DESeq2* Wald test, adjusted *P*-value < 0.05) in cell types within the duodenum of aSI-L compared to aSI-H adult female mice that are homologues of human proteins whose levels were significantly higher in women with EED (histopathology score 1-3) compared to healthy women without EED (histopathology score 0, *limma* adjusted *P*-value < 0.05). **(B)** Genes that were significantly more highly expressed (*DESeq2* Wald test, adjusted *P*-value < 0.05) in aSI-H compared to aSI-L adult female mice that correspond to human proteins whose levels were significantly higher in healthy women (histopathology score 0) compared to women with EED (histopathology score 1-3, *limma* adjusted *P*-value < 0.05). For both panels, cell types from the single nucleus RNA-Seq dataset were included if more than 100 nuclei were present, n=4 mice/group, • *P*-adj < 0.05.

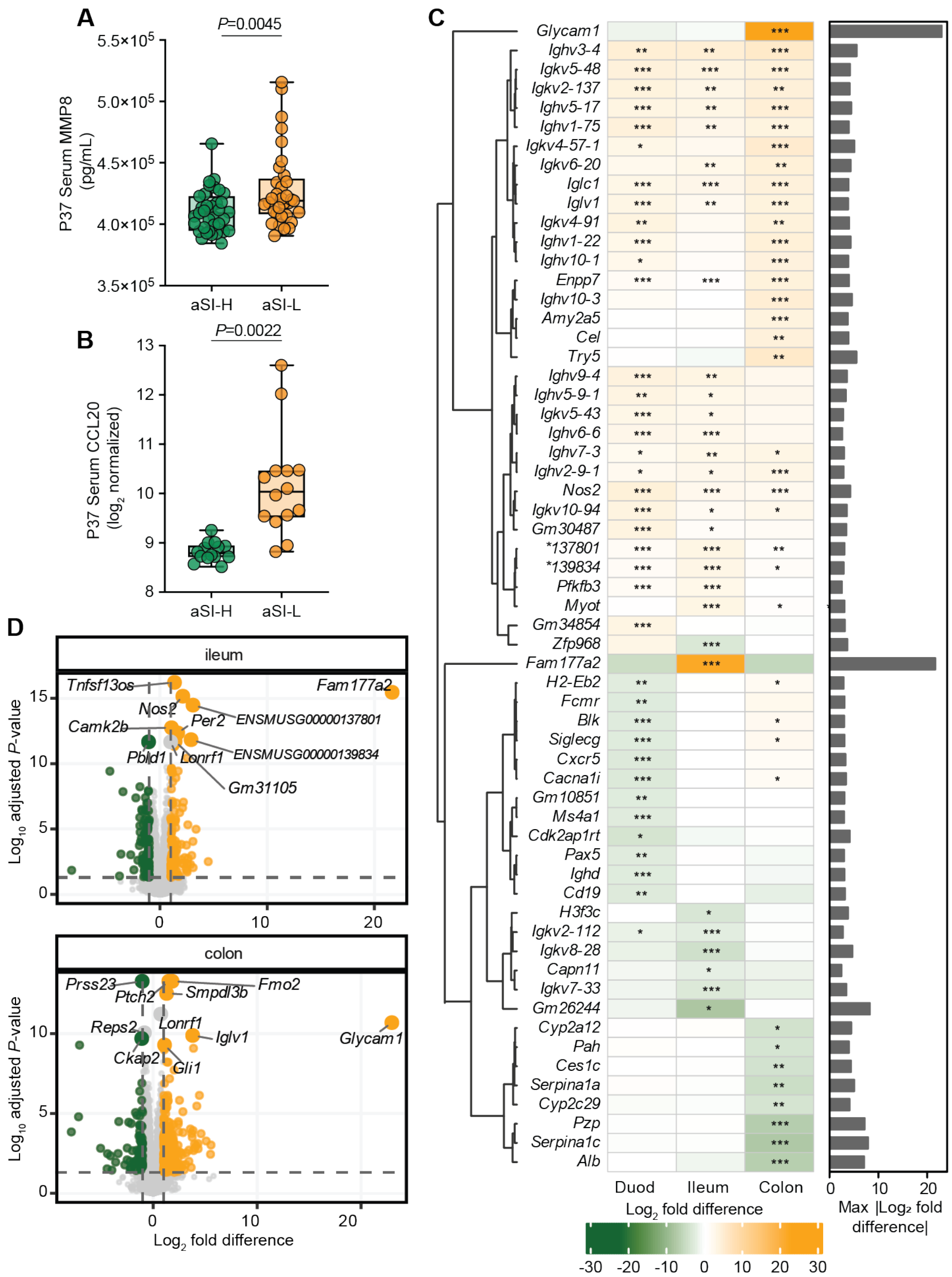

Fig. S6. Intestinal transcriptional analysis of P37 offspring. (A,B) Serum levels of MMP8 (A) and CCL20 (B). **(C,D)** Genes most significantly differentially expressed between aSI-L (orange, positive $\log_2$-fold difference) and aSI-H (green, negative) offspring in the duodenum, ileum, and colon. **(C)** Each column of the heatmap shows pairwise comparisons for each intestinal segment (*DESeq2* Wald test, * *P*-adj < 0.05, ** *P*-adj < 0.01, *** *P*-adj < 0.001). Bar chart annotation shows the maximum absolute $\log_2$-fold difference for each gene. **(D)** Volcano plots depicting the most significantly expressed genes in the ileum and colon (*DESeq2* Wald test). For (A), n=35 mice/group, unpaired two-sided t-test. For (B-D), n=14-16 animals/group; For (B), Benjamini Hochberg-corrected Wilcoxon rank-sum test.

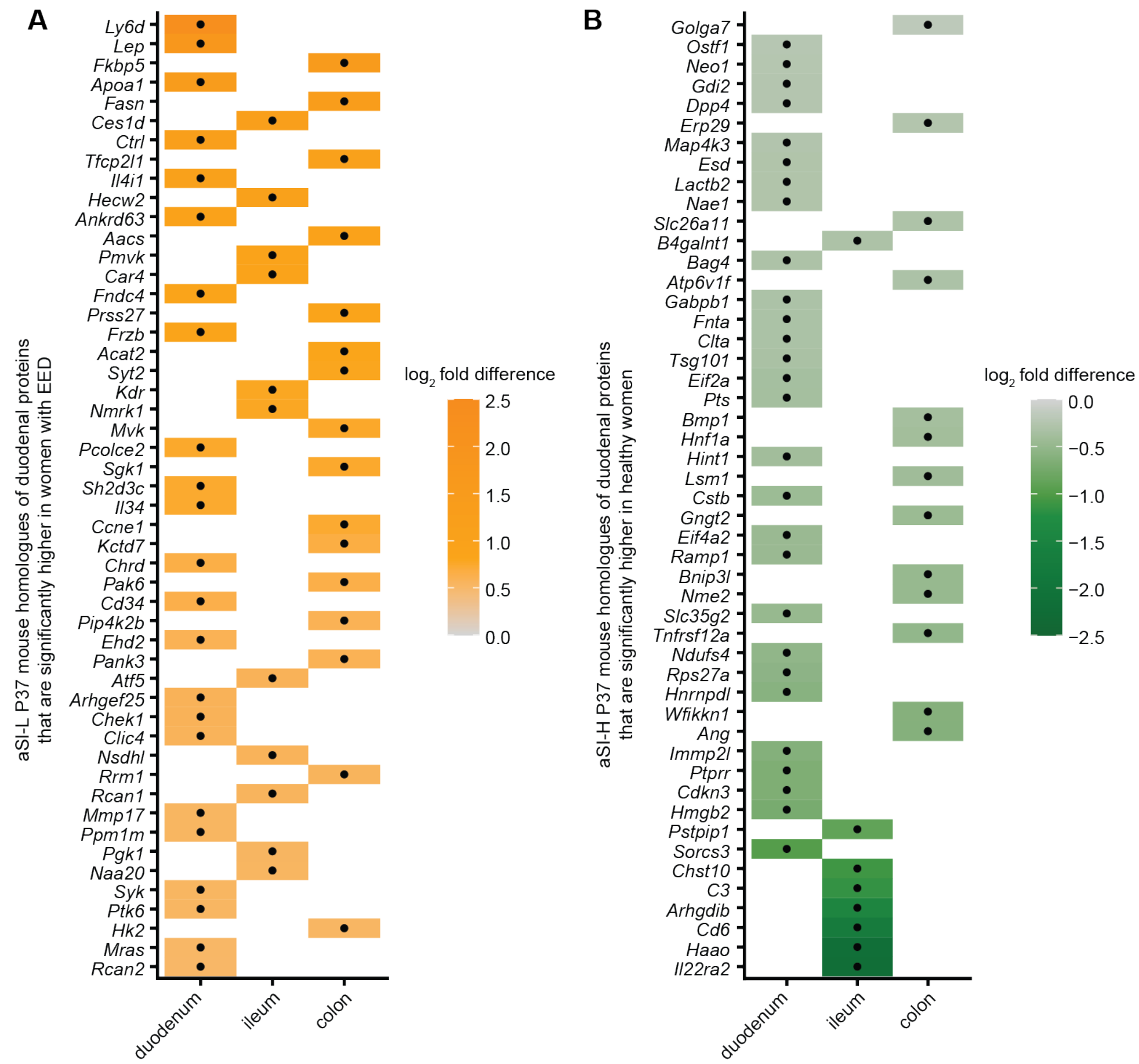

Fig. S7. Expression in P37 intestinal tissue of mouse genes that encode homologues of proteins whose levels were significantly different between women with and without EED. **(A,B)** Mouse genes that were significantly more highly expressed in aSI-L compared to aSI-H (A) or aSI-H compared to aSI-L (B) P37 offspring (*DESeq2* Wald test, • *P*-adj < 0.05).

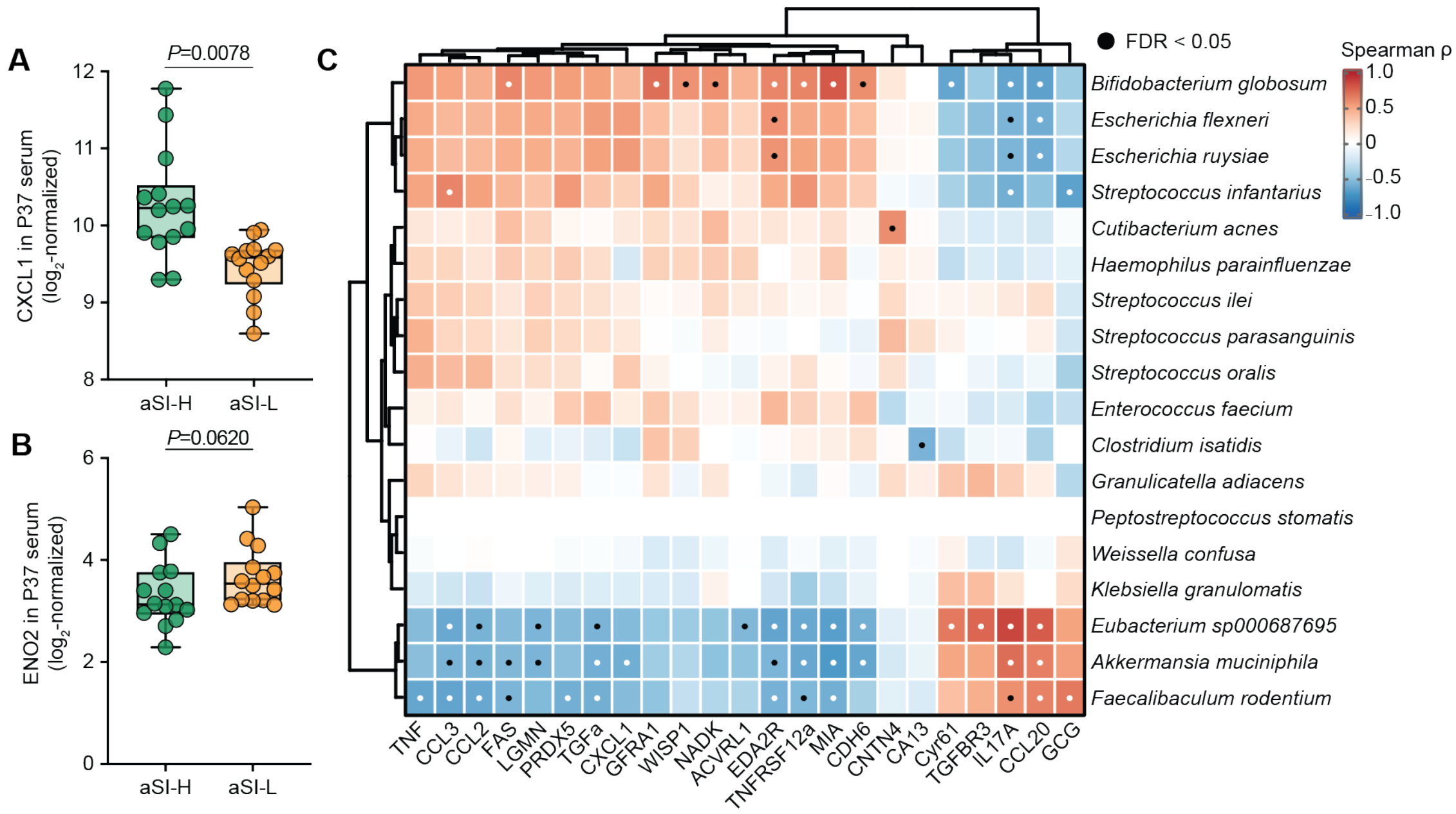

Fig. S8. Serum proteomic analysis of P37 offspring. (A,B) Serum levels of CXCL1 (A) and ENO2 (B) (Benjamini Hochberg-adjusted Wilcoxon rank-sum tests). **(C)** Correlations between absolute abundances of bacteria in cecal contents and levels of proteins in serum (FDR-corrected Spearman correlations, • *P*-adj < 0.05). For all panels, n=14 mice/group.

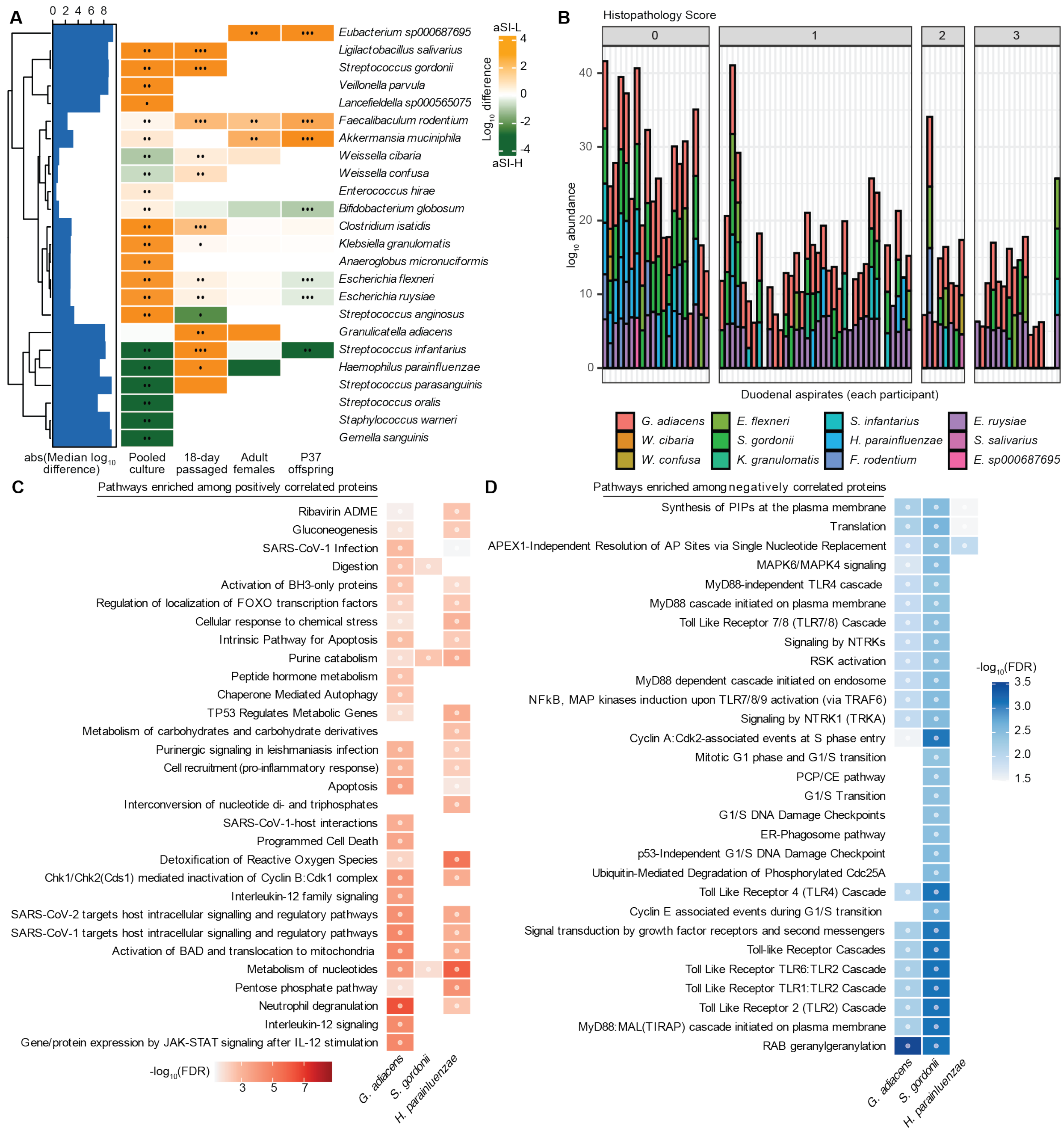

Fig. S9. Correlations between aSI-L taxa in duodenal aspirates with duodenal mucosal proteins. (A) Species whose absolute abundances were significantly different between aSI-L and aSI-H mice: 18-day post-weaning colonization with pooled culture collections and passaged LCN2-high and LCN2-low consortia, adult females, and P37 offspring. The left annotation shows the maximum difference in log_{10} median absolute abundance (Benjamini-Hochberg corrected Wilcoxon rank-sum tests; • *P*-adj < 0.1, •• *P*-adj < 0.05, ••• *P*-adj < 0.01). **(B)** Log_{10}-transformed absolute abundances of aSI-L bacteria in each duodenal aspirate (n=80 women). Aspirates are binned according to histopathology score. **(C,D)** Enrichment analysis was performed on duodenal proteins that were significantly positively (C) or negatively (D) correlated (FDR-corrected Spearman) with the absolute abundances of aSI-L bacterial taxa in the aspirates of women (• *P*-adj < 0.05).

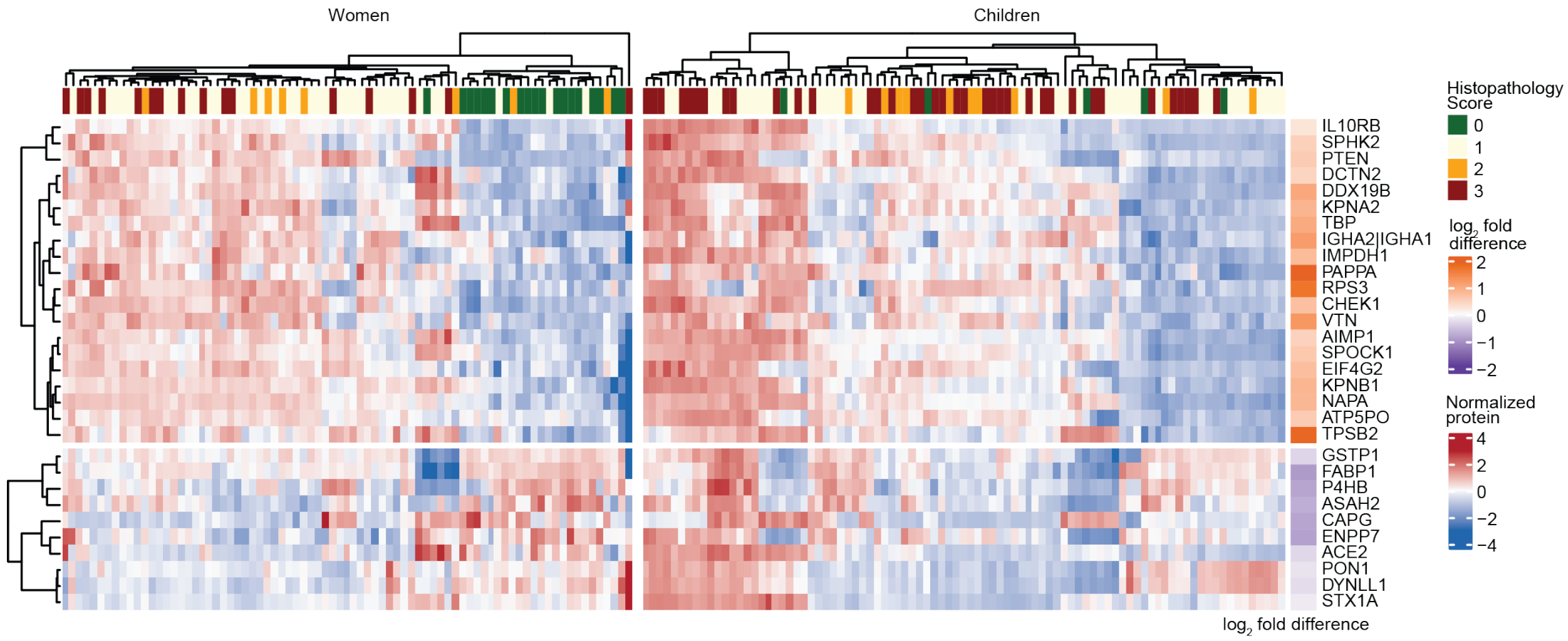

Fig. S10. Duodenal mucosal proteins whose levels differ between women and children with and without EED. Duodenal mucosal proteins that were the most significantly different between women and children with severe EED (histopathology score 3, n=16/79 women in the current study, 37/89 children in the BEED study who were undernourished and underwent EGD (*14*)) compared to those without evidence of enteropathy (histopathology score 0; n=20/79 women, 5 of the 89 children (*14*); *limma* empirical Bayes moderation). Protein levels are row-normalized within each cohort; the top annotation denotes histopathology score, and the right row annotation shows the $\log_2$ fold-difference across both women and children (n=79 biopsies from women, n=89 from children).
