# Supplementary material for: Functional characterization of duodenal microbiota and associated enteropathy in undernourished Bangladeshi women and gnotobiotic mice
Source: medRxiv. 2026 Sep 4:2026.08.14.26360472. Preprint. [Version 2] doi: 10.64898/2026.08.14.26360472 (PMC13684689; doi:10.64898/2026.08.14.26360472)
Supplement: Supplementary file 2 [file media-12.pdf]

SUPPLEMENTARY RESULTS

Duodenal bacterial taxa and duodenal proteins predictive of EED status

We utilized a supervised learning approach, 'Data Integration Analysis for Biomarker discovery using Latent variable approaches for 'Omics' (DIABLO; a 'block' sparse partial least-squares discriminant analysis), to find predictive bacterial and proteomic features of EED (**fig. S11A,B**) (*62*). The overall balanced error rate for the DIABLO model was 0.1179, with individual class error rates of 0.1008 for levels of duodenal mucosal proteins and 0.1101 for bacterial abundances in duodenal aspirates. Duodenal mucosal proteins that were most predictive of EED (histopathology score ≥1) included Tyrosine Kinase (SYK), which mediates inflammatory responses at the intestinal epithelium and multiple leukocyte lineages; Ring Finger Protein 31 (RNF31), which activates NF-kB; as well as proteins involved in proliferation [Eukaryotic Elongation Factor 2 Kinase (EEF2K), Deoxyhypusine Hydroxylase (DOHH)] and DNA repair [8-Oxoguanine glycosylase (OGG1), Polynucleotide Kinase 3'-Phosphatase (PNKP)] (**fig. S11C, table S3A)**. Proteins that were associated with a lack of pathology (histopathology score 0) included two that promote cytoskeletal organization and cell signaling, Osteoclast Stimulating Factor 1 (OSTF1) and Tubulin Polymerization Promoting Protein Family Member 3 (TPPP3), as well as Dual Specificity Phosphatase 3 (DUSP3), which dephosphorylates mitogen-activated protein kinases (MAPKs) (**fig. S11C, table S3A**). Duodenal bacteria most predictive of the absence of pathology included *Bulleidia timonensis*, *Lancefieldella rimae*, *Lachnoanaerobaculum orale*, *Anaeroglobus micronuciformis* and *Rothia aeria* (**figs. S11D and S12 table S3B**). Integrating the absolute abundances of bacterial taxa in duodenal aspirates with levels of proteins in the duodenal mucosa predicts disease status and identifies potential therapeutic targets.

Associations between fecal bacterial taxa and the plasma proteome in undernourished compared to healthy women

Full-length 16S rRNA long-read sequencing identified 22,495 unique ASVs and 538 unique species in fecal samples from the Low BMI (n=38) and well-nourished (n=25) cohorts (**fig. S13A, table S4A**). After prevalence filtering for species present in 50% of the smallest cohort (n=12 subjects), 175 unique species remained and were included in subsequent analysis. The relative abundances of 59 taxa were significantly different between the Low BMI and Normal BMI cohorts (**fig. S13B**, Benjamini Hochberg-adjusted Wilcoxon rank-sum*,* adjusted *P*-value<0.05). The relative abundances of members of the genus *Prevotella* (*Prevotella pectinovora, Prevotella spp001553265, Prevotella sp003447235*), *Treponema succinifaciens*, *Succinivibrio sp000431835*, as well as members of the family Oscillospiraceae (*Faecousia sp000434635*, *Vescimonas fastidiosa*) and *Cryptobacteroides* were significantly higher in undernourished women (**table S4B**), while the relative abundances of *Bifidobacteria* (*Bifidobacterium longum, Bifidobacterium pseudocatenulatum,* and *Bifidobacterium faecale*), *Sutterella wadsworthensis*, *Streptococcus thermophilus* and *Parabacteroides distastonis* were significantly higher in the feces of healthy women (**table S4B**). In addition, we quantified the burden of common enteropathogens in DNA extracted from fecal samples using a microfluidics-based quantitative PCR platform and found that the burden of enteropathogens was higher in women who were part of the Low BMI cohort (**fig. S13C, table S4C**).

We used Data Integration Analysis for Biomarker discovery using Latent components (DIABLO) (*62*) to identify the best plasma protein and fecal bacterial predictors of nutritional status. The plasma proteome strongly differentiates undernutrition in this Bangladeshi cohort (individual class error 0.0008696), while fecal bacterial composition predicts undernutrition less successfully (individual class error 0.2404, overall balanced error rate 0.0008, **fig. S4A,B**). Plasma proteins that were the strongest predictors of undernutrition in women included: complement component 3 (C3), Chromosome 11 Open Reading Frame 52 (C11orf52, cell-cell junctions), Kinesin-like protein (KIF1A, axonal transporter of synaptic vesicles), Thymidylate Synthetase Opposite Strand (TYMSOS, cellular proliferation), Nucleotide-Binding Protein 2 (NUBP2, iron-sulfur cluster assembly factor) and Small Proline-Rich Protein 3 (SPRR3, epithelial barriers) (**fig. S14C**, **table S5A**). *Mogibacterium kristiansenii, Cryptobacteroides sp00435075, Prevotella pectinovora,* and *Hominicoprocola sp900317525* were the best predictors of low BMI, while *Bifidobacterium faecale* was the best predictor of healthy BMI (**fig. S14D**, **table S5B**).

As with the abundances of duodenal bacteria and duodenal mucosal proteins, we performed linear regressions on the relative abundances of fecal bacteria and levels of plasma proteins. This time, we sought bacteria-protein pairs whose slopes were significantly different in women who were undernourished (Low BMI cohort) compared to healthy women (Normal BMI cohort, *lmFit* followed by *eBayes*, see *Methods*). A *Prevotella* strain exhibited the greatest number of significant associations with plasma proteins (**fig. S5A**), followed by *Enterobacter cloacae* and *Catenibacterium mitsuokai*. The abundances of several immuno-inflammatory plasma proteins were positively linearly related to the abundances of fecal bacteria across both cohorts of women, including Nitric Oxide Synthase 1 Adaptor Protein (NOS1AP), Interleukin-17C (IL-17C), and Interferon-Lambda 4 (IFNL4, **fig. S15B**, **table S5C**). Conversely, plasma proteins that were negatively correlated included Fibroblast Growth Factor 8 (FGF8), Transforming growth factor beta-3 (TGFB3) and SRY-box transcription factor 7 (SOX7, **fig. S15B**, **table S5C**).

In total, 2,579 plasma proteins exhibited statistically significant linear relationships with the abundances of 92 fecal bacteria that were positively associated in members of the Low BMI cohort, while negatively or neutrally associated with members of the Normal BMI cohort (**table S5C**). The bacteria-plasma protein pairs that displayed the largest magnitude of difference between undernourished and healthy women included *S. parasanguinis* with Suprabasin (SBSN) and Huntingtin-interacting protein (HIP1), two proteins involved in cancer progression, *Enterobacter quasiroggenkampii* and *Weisella confusa* with Lymphocyte Antigen 86 (LY86), which forms a complex with CD180 to mediate antibacterial immune responses, as well as *Ruminococcus gnavus* with Homocysteine-responsive endoplasmic reticulum-resident ubiquitin-like domain member 2 protein (HERPUD2), which plays a key role in cellular stress responses (**table S5C**). Several interleukins, complement cascade proteins, C-type lectin proteins (CLEC), Carcinoembryonic antigen-related cell adhesion molecules (CEACAMs), chemokine C-X-C motif ligand proteins (CXCL family), interferons and caspases were similarly positively associated with the abundances of fecal bacteria in undernourished but not healthy women (**table S5C**). We performed functional enrichment analyses on this set of plasma proteins whose slopes were significantly higher in undernourished women compared to healthy women (1,563 proteins and 101 bacteria, **fig. S16A**). These plasma proteins were involved in cell surface interactions at the vascular wall, chromatin and histone modifications, epigenetic modifications including DNA methylation, and DNA repair (**fig. S16A, table S5D**).

Conversely, there were 994 plasma proteins that were positively linearly related with the relative abundances of 90 bacterial taxa in women that were part of the Normal BMI cohort, but negatively or neutrally associated in members of the low BMI cohort (**table S5C**). These bacteria-plasma protein included *Mogibacterium kristiansenii* with (i) Misshapen-like kinase 1 (MINK1), a central regulator of several cellular processes including the Hippo/JNK/p38 pathways, (ii) the Prolactin Receptor (PRLR) and (iii) AXL, a receptor tyrosine kinase that is a negative regulator of inflammation. This set of plasma proteins that exhibited negative or neutral relationships with the abundances of fecal bacteria in low BMI women and positive associations in healthy women were involved in one carbon/folate metabolism, cysteine and methionine metabolism, p53 stabilization and lipoprotein binding (**fig. S16B, table S5E**). Together, these analyses identified bacteria in the distal gut and their associations with the plasma proteome that distinguish nutritional status in these cohorts of Bangladeshi women.

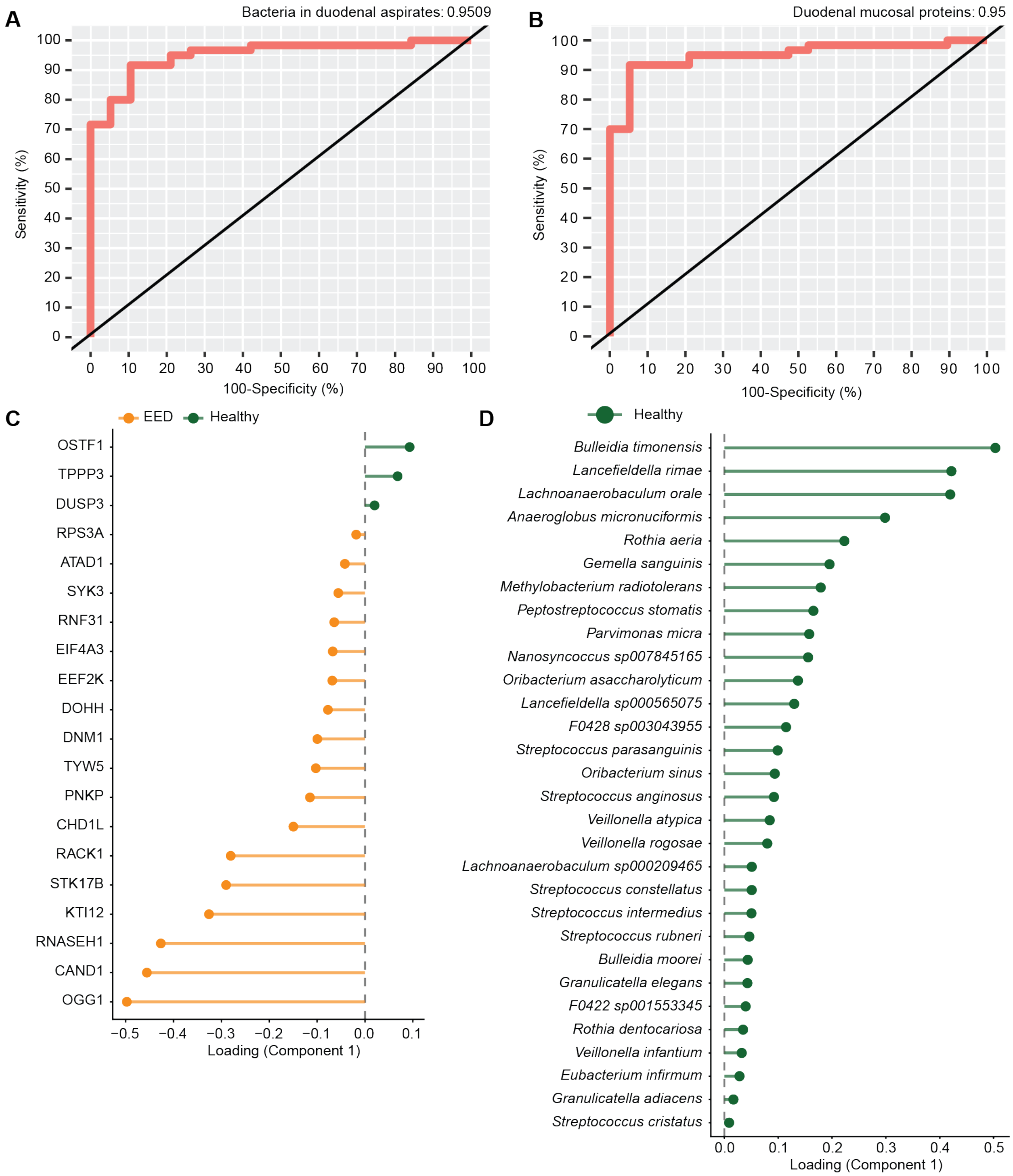

Fig. S11. Feature prediction for DIABLO analysis. (A,B) Area Under the Receiver Operating Characteristic (AUROC) plots depicting the individual performance of the binary classification models for bacteria in duodenal aspirates (A) and proteins in duodenal biopsies (B), which were both part of the combined DIABLO model. **(C,D)** Proteins (C) and bacteria (D) in duodenal mucosal biopsies and aspirates, respectively, that differentiate women with EED (histopathology score 1-3, orange, n=59 out of 79 women) from healthy women (green, histopathology score 0, n=20 out of 79 women).

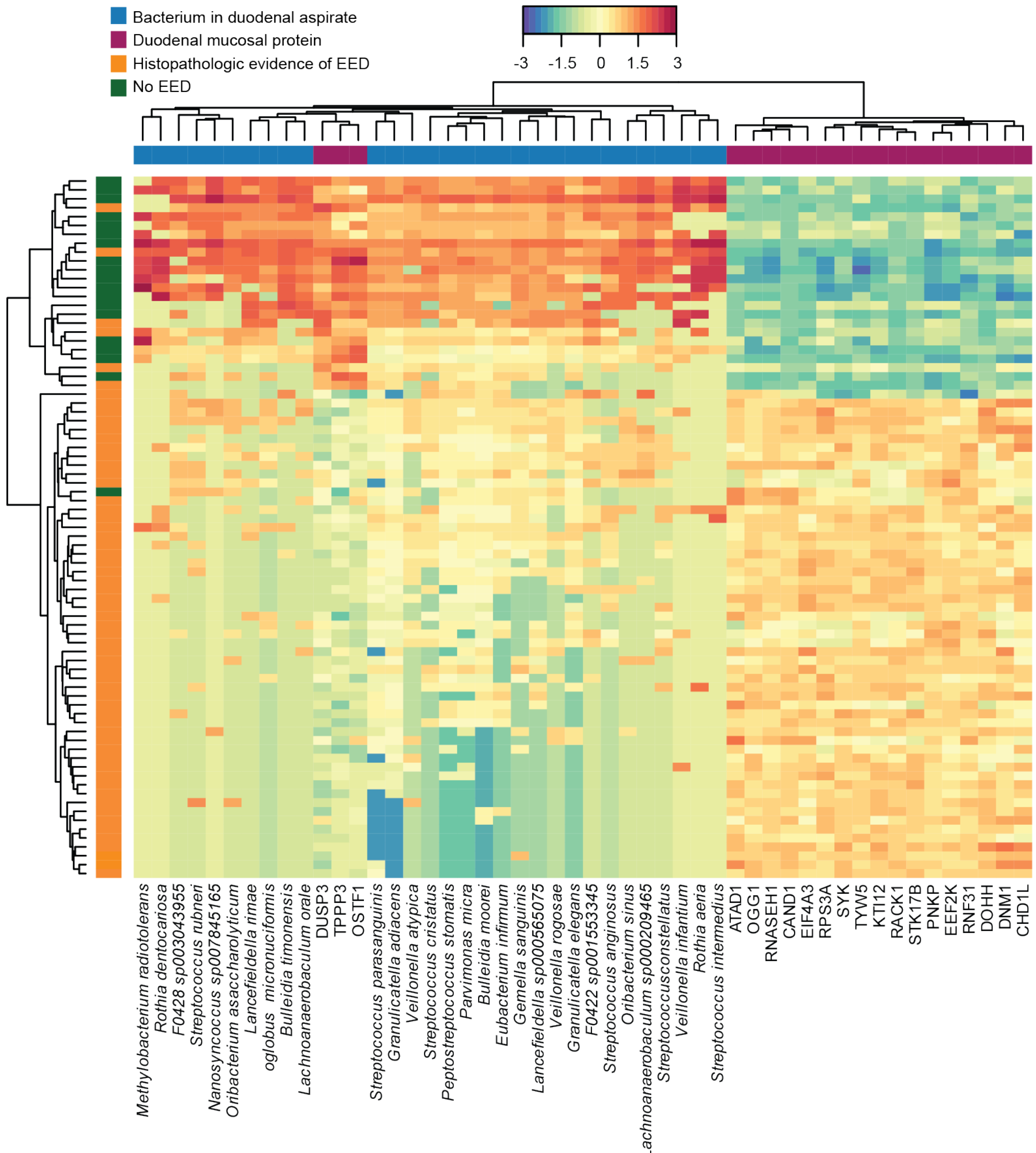

Fig. S12. Duodenal bacteria and mucosal proteins that predict disease status. Heatmap depicting discriminatory features contributing to DIABLO analysis integrating absolute bacterial abundance in aspirates with the duodenal mucosal proteome of women with EED (histopathology score ≥1) compared to healthy controls (histopathology score 0).

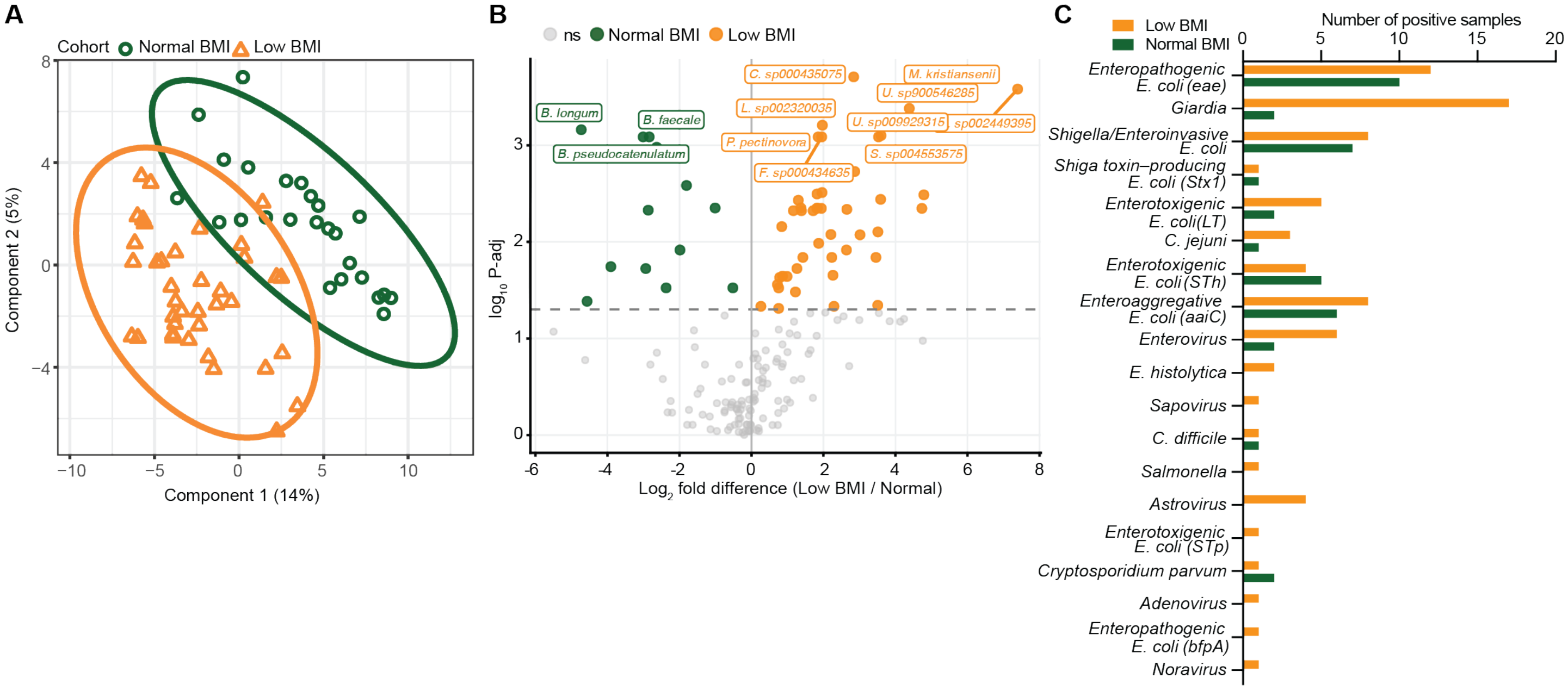

Fig. S13. Composition of fecal microbiota in Low BMI cohort compared to Normal BMI cohort. (A) Sparse partial least squares discriminant analysis (sPLS-DA) of the relative abundance of bacterial species in fecal samples from undernourished compared to healthy donors. Ellipses denote 95% confidence intervals. **(B)** Bacteria whose relative abundances are significantly higher in undernourished women (orange, positive) compared to healthy women (normal BMI, green, negative; FDR-corrected wilcoxon rank-sum tests). **(C)** Prevalence of common enteropathogens in feces of undernourished and healthy women detected with a microfluidics qPCR-based platform.

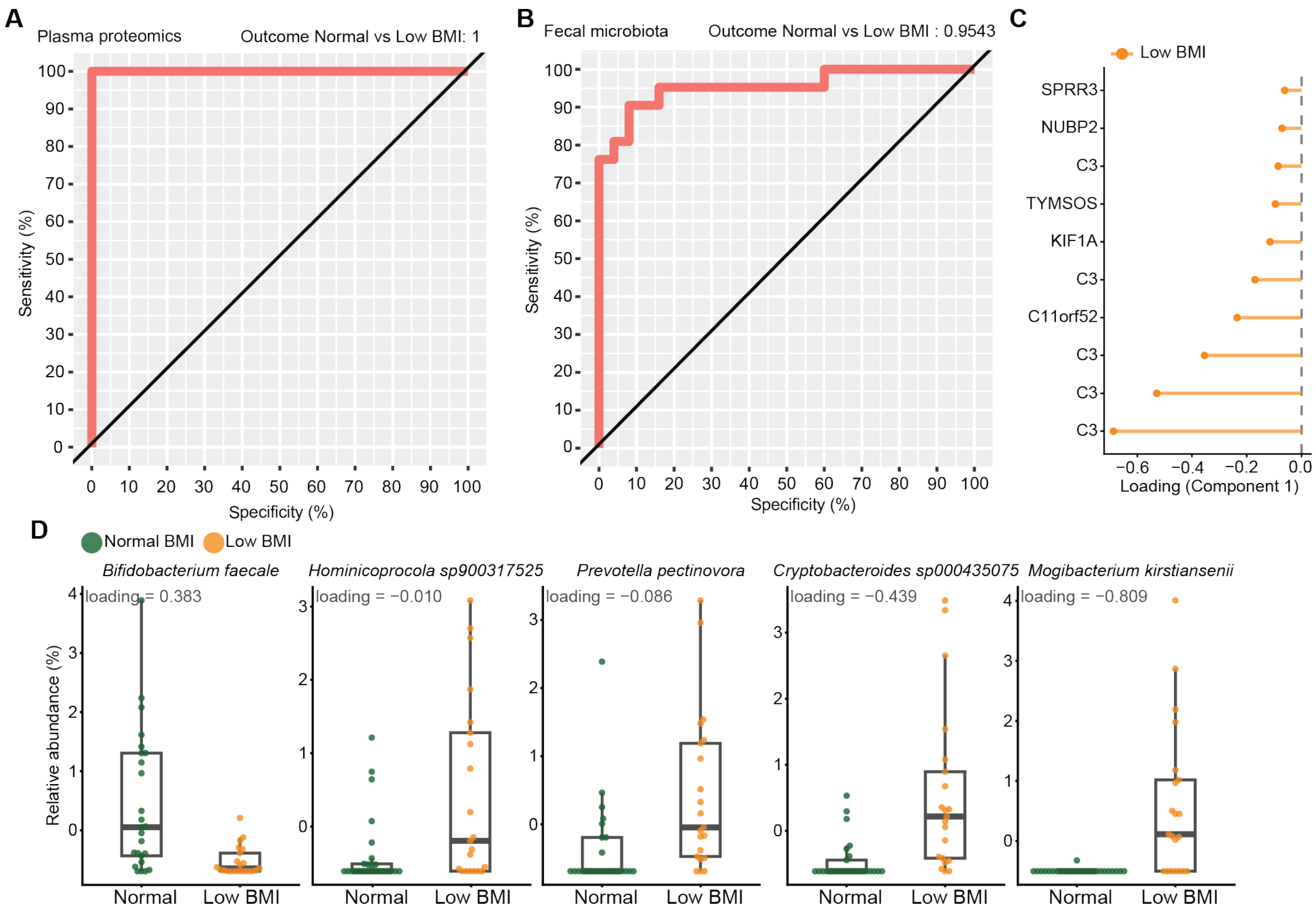

Fig. S14. Plasma proteins and fecal bacteria that predict nutritional status. (A,B) Area Under the Receiver Operating Characteristic (AUROC) plots depicting the individual performance of the binary classification models for plasma proteins (A) and bacteria in fecal specimens (B) which together formed the combined DIABLO model. **(C,D)** The most discriminant plasma proteins (C) and fecal bacteria (D) that predict cohort (DIABLO).

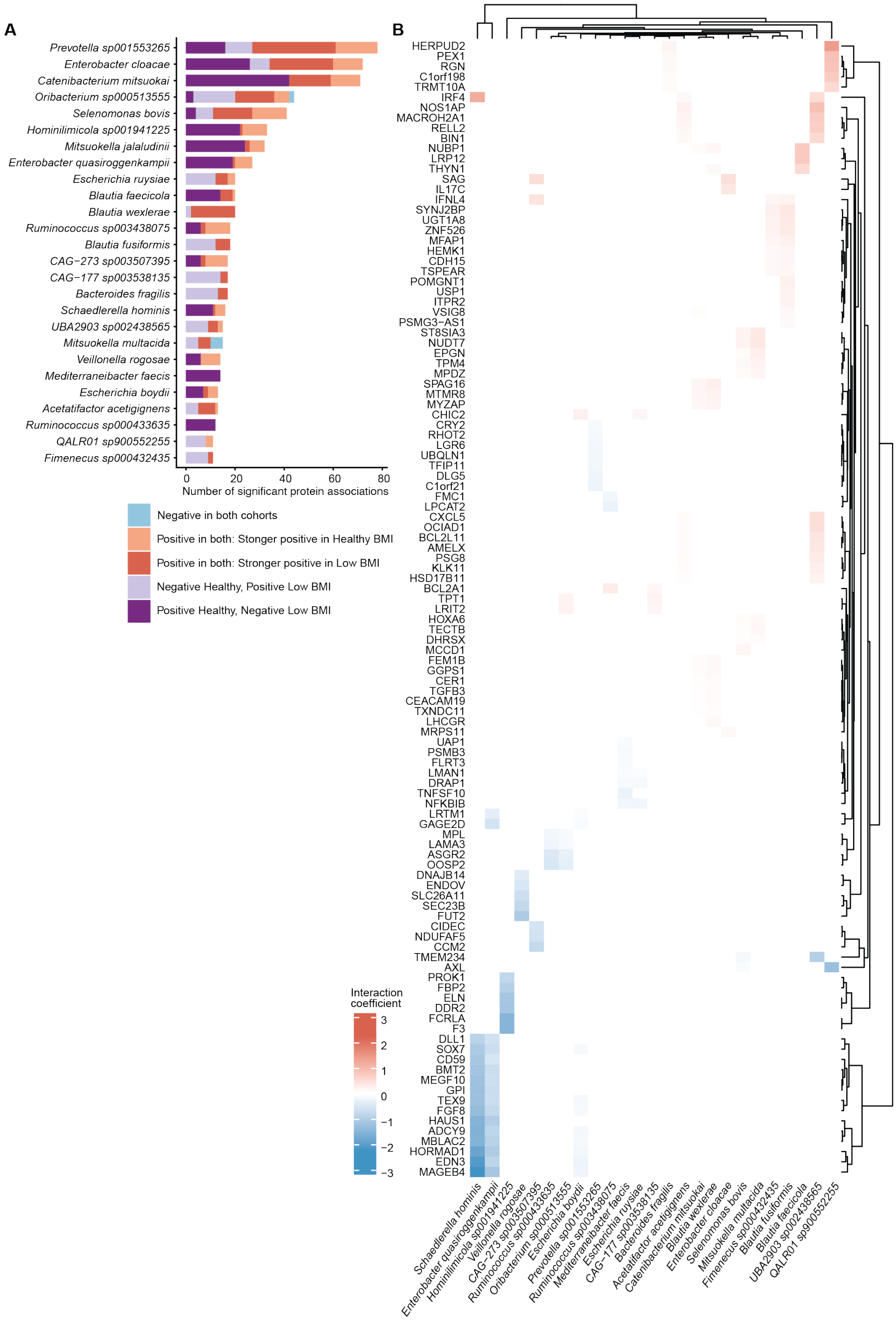

Fig. S15. Linear models associating plasma protein levels with abundances of fecal bacteria. (A) Bacteria in fecal samples that exhibited the greatest number of significant associations with plasma proteins in women and how the relationships with plasma proteins differ between undernourished women (low BMI) compared to healthy controls (healthy BMI). **(B)** Heatmap showing the ß coefficients (slopes) for the most significant results of linear models relating fecal bacterial abundance to plasma protein levels across all women.

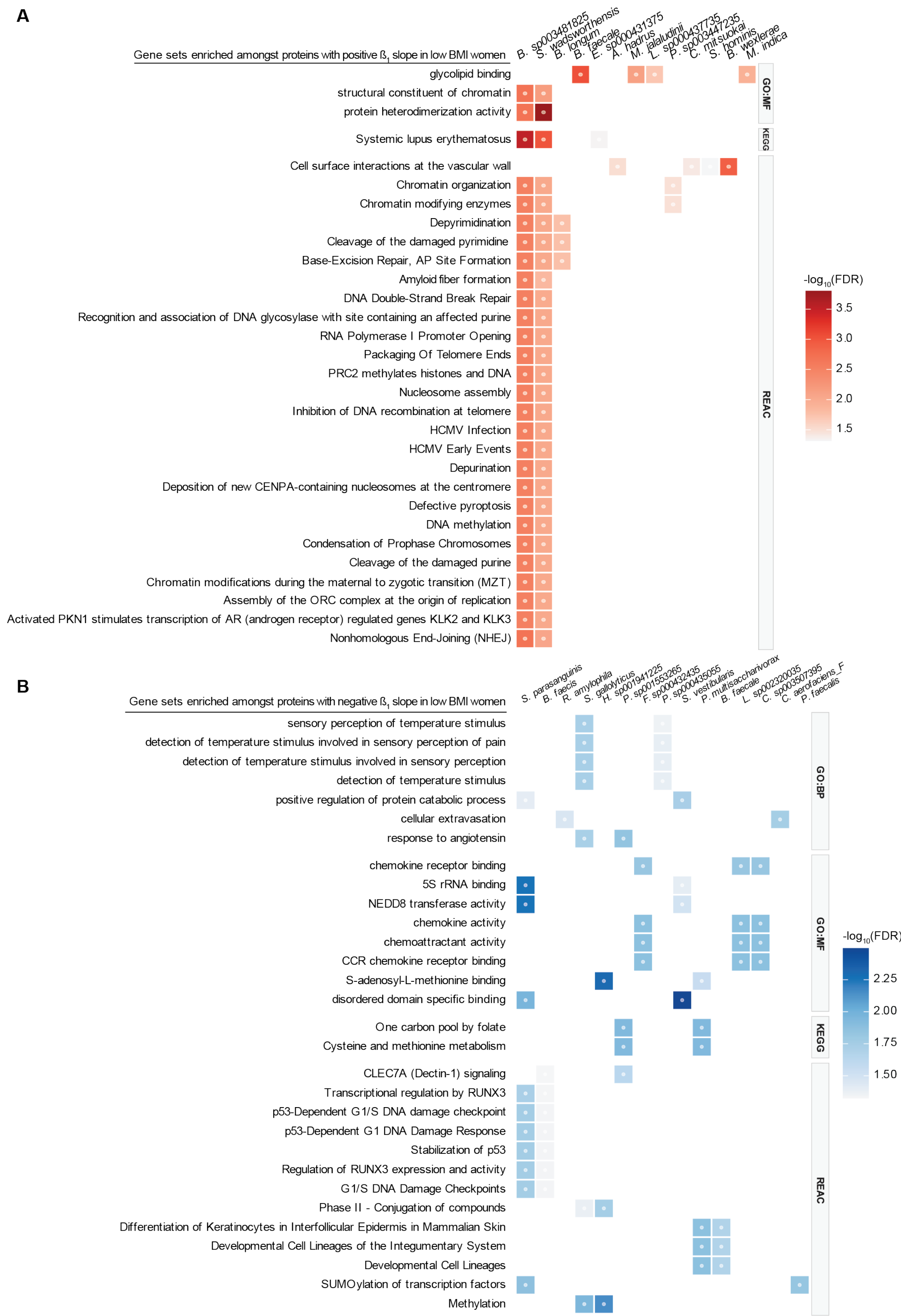

Fig. S16. Functional enrichment analysis of plasma proteins signficantly associated with the abundance of fecal bacteria. (A) Statistical enrichment analysis of plasma proteins that were significantly positively linearly related to bacterial abundance in feces in undernoursiehd women, while negatively or neutrally associated in healthy controls. **(B)** Enrichment analysis of plasma proteins that were negatively associated with bacterial abundance in fecal samples from undernurished women, while positive or neutral in healthy women. For both A and B, Gene Ontology (GO) categories, KEGG and Reactome pathway databases were queried for the results of linear models.

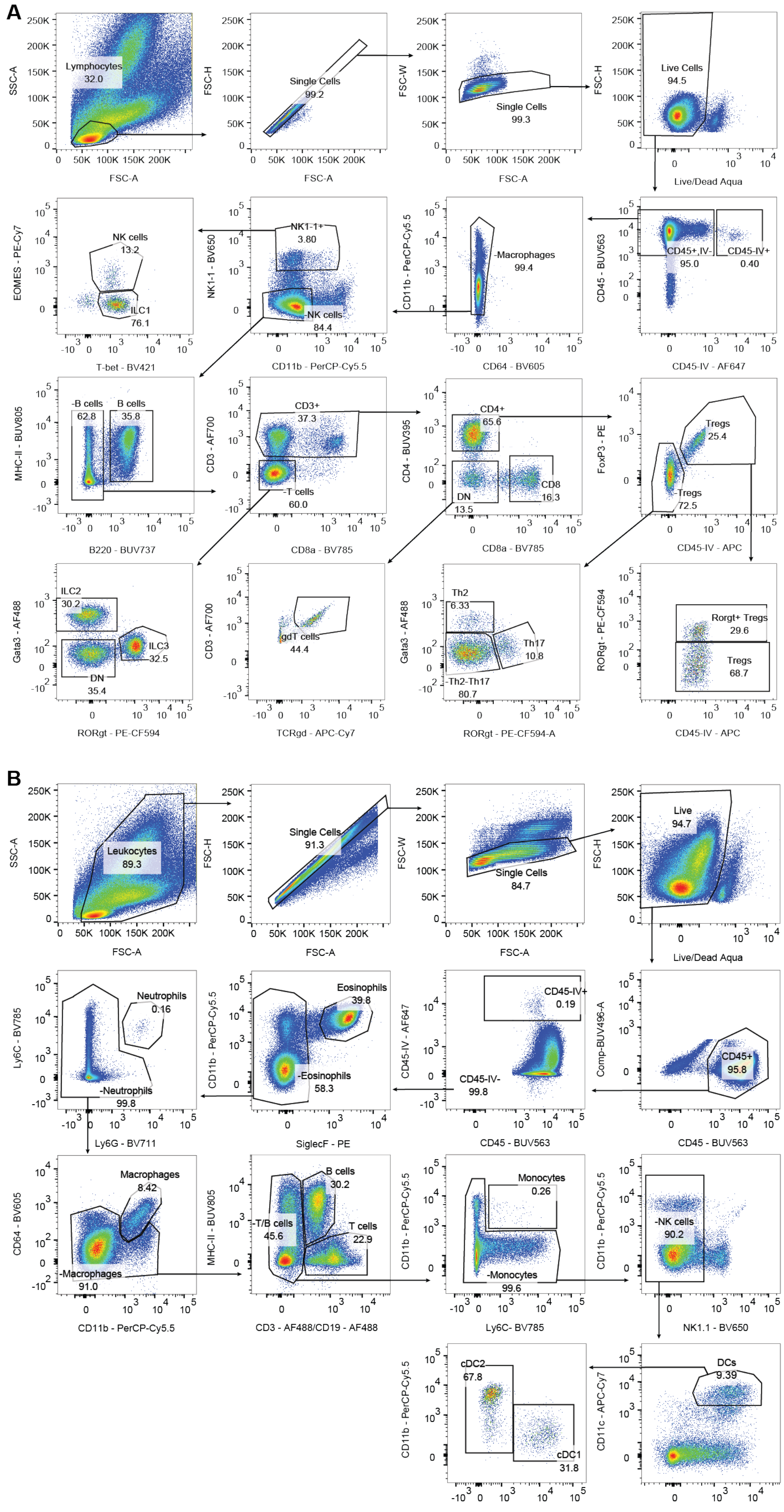

Fig. S17. Gating schemes used in flow cytometry analysis. (A) Gating strategy for identification of lymphoid populations. **(B)** Gating strategy for identification of myeloid populations.
